# Identification and Validation of a Prognostic Model Based on Tumour Necrosis Factor‐Related mRNAs for Kidney Renal Clear Cell Carcinoma

**DOI:** 10.1111/jcmm.70657

**Published:** 2025-07-17

**Authors:** Zijian Hu, Yajie Zhou, Lei Xie, Shuwen Zhang, Yijiang Liu, Wenxiong Zhang, Ting Huang

**Affiliations:** ^1^ Department of Thoracic Surgery, The Second Affiliated Hospital, Jiangxi Medical College Nanchang University Nanchang China; ^2^ Department of Urology Surgery, The Second Affiliated Hospital, Jiangxi Medical College Nanchang University Nanchang China; ^3^ Jiangxi Medical College Nanchang University Nanchang China; ^4^ Department of Blood Transfusion, The Second Affiliated Hospital, Jiangxi Medical College Nanchang University Nanchang China

**Keywords:** kidney clear cell carcinoma, messenger RNA, prognostic model, tumour necrosis factor

## Abstract

Tumour necrosis factor (TNF) plays a critical role in tumour progression, but the specific involvement of mRNA in this process, particularly in kidney renal clear cell carcinoma (KIRC) remains insufficiently understood. Our study aims to develop a TNF‐related mRNA (TRmRNA) model to predict prognosis and inform treatment strategies in KIRC. KIRC expression data from The Cancer Genome Atlas (TCGA) and TNF‐related genes (TRGs) from the Genecards database were used to construct and validate a TRmRNA prognostic model. A nomogram integrating clinical features with the risk model was also developed to enhance prognostic accuracy. Enrichment analysis, drug sensitivity analysis and RT‐qPCR validation were performed to further explore the biological mechanisms and clinical applicability of the model. A prognostic signature consisting of nine TRmRNAs was identified. Kaplan–Meier analysis showed that the high‐risk (HRK) group had significantly shorter overall survival (OS) compared to the low‐risk (LRG) group (*p* < 0.001). The nomogram, incorporating the risk model, yielded an area under the curve (AUC) of 0.766, indicating robust prognostic accuracy. Enrichment analysis identified solute sodium symporter and proximal tubule transport pathways enriched in the LRG group, whereas the HRK group exhibited enrichment in CD22‐mediated BCR regulation and immunoglobulin complex pathways. The HRK group also showed a higher tumour mutational burden (TMB), correlating with a poorer prognosis. RT‐qPCR confirmed the differential expression of mRNAs in KIRC cells. The TRmRNA‐based prognostic model holds significant promise for predicting patient outcomes and guiding personalised treatment strategies in KIRC.

**Abbreviations**
AJCC: American Joint Committee on Cancer
APC: Antigen‐presenting cell
AUC: Area Under the Curve
DCA: Decision Curve Analysis
EMT: Epithelial‐mesenchymal transition
FDR: False Discovery Rate
GSEA: Gene Set Enrichment Analysis
HR: Hazard Ratios
HR: High risk
IC50: Half‐Maximal Inhibitory Concentrations
ICIs: Immune checkpoint inhibitors
KEGG: Kyoto Encyclopedia of Genes and Genomes
KIRC: Kidney Renal Clear Cell Carcinoma
LASSO: Least Absolute Shrinkage and Selection Operator
LR: Low risk
LUAD: Lung Adenocarcinoma
mCox: Multivariate Cox regression
mRNA: Messenger RNA
OS: Overall Survival
PCA: Principal Component Analysis
PDGFRβ: Platelet‐derived growth factor receptor β
PPI: Protein–Protein Interaction
ROC: Receiver Operating Characteristic
RT‐qPCR: Quantitative Reverse Transcription Polymerase Chain Reaction
ssGSEA: Single‐Sample Gene Set Enrichment Analysis
STAD: Stomach Adenocarcinoma
TCGA: The Cancer Genome Atlas
TIDE: Tumour Immune Dysfunction and Exclusion
TIMER: Tumour Immune Estimation Resource
TMB: Tumour Mutational Burden
TME: Tumour Microenvironment
TNF: Tumour Necrosis Factor
TNM: Tumour‐Node‐Metastasis
TRGs: TNF‐related genes
TRmRNA: TNF‐related mRNA
uCox: Univariate Cox regression
UCSC: University of California, Santa Cruz

## 1 Introduction

Kidney renal clear cell carcinoma (KIRC) represents one of the most prevalent urogenital cancers, with the incidence of this cancer rising by 2%–3% each year [1]. The tumour‐node‐metastasis (TNM) classification by the American Joint Committee on Cancer (AJCC) is a common method to evaluate disease progression for prognosis [2]. However, this staging system has notable limitations in accurately predicting patient outcomes, reducing its utility in optimising treatment strategies [3]. Consequently, the demand for accurate and dependable prognostic models to support clinical decisions and enhance KIRC patient management has become increasingly urgent. In recent years, biomarker‐based models have gained attention as a promising tool, offering improved accuracy and precision in cancer prognosis assessment.

The tumour necrosis factor (TNF) family comprises 19 ligands and 29 receptors, both of which are significant in cancer progression [4]. Previous research has demonstrated that signatures based on the TNF family can serve as effective prognostic tools and predict the response to immunotherapy and chemotherapy in various cancers, such as lung adenocarcinoma (LUAD) and colorectal cancer [5, 6]. Moreover, TNF and its receptors are emerging as promising therapeutic targets for treating rheumatic and other inflammatory diseases [7]. Therefore, the TNF family represents a promising avenue for enhancing cancer prognosis and developing novel immunotherapeutic strategies. At the same time, messenger RNA (mRNA) has become an essential component in cancer prognosis, with numerous mRNA‐based prognostic models successfully developed for different types of cancer. For example, Zou et al. established an mRNA‐based prognostic model for cervical squamous carcinoma and adenocarcinoma, which was validated for identifying precise and effective biomarkers [8]. Likewise, Jiang et al. proposed an mRNA‐based model for stomach adenocarcinoma (STAD) that provided reliable predictions of clinical outcomes and targeted therapy responses [9]. However, to date, no research has focused on developing a prognostic model for KIRC based on TNF‐related mRNA (TRmRNA).

Building upon this foundation, a predictive model using TRmRNAs was developed and validated to explore its underlying mechanisms and evaluate its clinical relevance. This was accomplished by performing a range of analyses, such as enrichment analysis, assessment of tumour mutational burden (TMB), profiling of the tumour microenvironment (TME) and predictions of drug sensitivity.

## 2 Materials and Methods

### 2.1 Data Acquisition and Organisation

Data for our research were primarily collected from the University of California, Santa Cruz (UCSC) Xena database (https://xena.ucsc.edu/, available until July 1st, 2024) [10]. We collected KIRC transcriptional data for 607 cases and clinical data for 979 cases from The Cancer Genome Atlas (TCGA) database via Xena. Our study included only patients who had complete clinical and transcriptional data available from datasets. In total, 598 samples were used for the further analysis. To reduce potential technical variability in sequencing depth and platform artefacts within TCGA data, we used FPKM‐normalised transcriptomic data downloaded via the UCSC Xena platform, which had undergone uniform preprocessing.

### 2.2 Select TNF‐Related Genes (TRGs) and TRmRNAs

TRGs were sourced from the GeneCards database, which were subsequently selected for further analysis [11]. To investigate the relationship between TRGs and all mRNAs, Pearson correlation analysis was conducted (|correlation coefficient| > 0.3 and *p* < 0.05), aiming to identify TRmRNAs. To further refine the results, variance analysis was conducted through the “DESeq2” package to filter mRNAs (*p* < 0.05 and |log2‐fold change| > 1.2) [12].

Subsequently, we split the KIRC patient data into train and test groups at a 1:1 ratio. TRmRNAs linked to survival were detected through univariate Cox (uCox) analysis. TRmRNAs strongly correlated with prognosis were pinpointed through the least absolute shrinkage and selection operator (LASSO) and multivariate Cox (mCox) analysis.

### 2.3 Establish TRmRNA Predictive Model

The findings were used to develop a predictive model, with the risk score determined by the following way: risk score = (coefficient of mRNA1 × expression level of mRNA1) + … + (coefficient of mRNAn × expression level of mRNAn). Using the midpoint of the score, patients were divided into two groups: high‐risk (HR) and low‐risk (LR) groups. Kaplan–Meier (K‐M) analysis for these cohorts was formulated through the “survival” package in R to analyse overall survival (OS) differences between the HR and LR groups. The “timeROC” package in R was employed to evaluate the model's predictive ability over 1, 3 and 5 years by generating Receiver operating characteristic (ROC) curves for the cohorts. Confidence intervals (95% CI) for AUC values were estimated via bootstrapping with 1000 iterations. The statistical significance of AUCs was assessed using permutation‐based *p*‐values.

Principal component analysis (PCA) was applied to examine the expression profiles of all genes, mRNAs, TRmRNAs and those included in the prognostic models, aiming to evaluate subgroup classification effectiveness. The uCox and mCox analyses were conducted to assess the independent prognostic capacity for our model, integrating risk scores with clinical variables. To further confirm the model's performance, K‐M survival analysis assessed its prognostic accuracy across multiple clinical subgroups.

### 2.4 Permutation Testing

To evaluate whether the predictive performance of the TRmRNA‐based prognostic model could have arisen by chance, we conducted permutation testing. We performed 1000 iterations, in each of which the overall survival status was randomly permuted across patients while maintaining the original TRmRNA expression matrix. For each permuted dataset, we applied the same LASSO and multivariate Cox regression pipeline to reconstruct the risk model and calculate its C‐index and AUC values. The distribution of performance metrics from the permuted models was then compared to that of the original model. A P‐value was calculated based on the proportion of permuted models whose performance exceeded that of the original model.

### 2.5 Creation of Nomogram to Predict OS

A nomogram was developed for a prognostic model, integrating clinical factors such as age, gender and stage to assess OS at 1, 3 and 5 years [13]. Additionally, we created a calibration plot that included the risk model and assessed the impact of the prognostic model on the consistency between survival prediction and actual outcomes by comparing its C‐index with that of the plot without the risk model. To compare the predictive power of the nomogram with that of the TNM stage alone, we performed statistical model comparisons using both the concordance index (C‐index) and the likelihood ratio test (LRT). Cox proportional hazards models were constructed using the coxph () and cph () functions in R, and the LRT was performed using the anova () function. A P‐value < 0.05 was considered statistically significant.

### 2.6 Enrichment Analysis

Based on outcomes derived from the expression of 9 TRmRNAs, the functional enrichment analysis was conducted to investigate functions linked to the identified genetic set, utilising Gene Set Enrichment Analysis (GSEA) 4.3.2 software. The P‐value was < 0.05 and the false discovery rate (FDR) was < 0.25 for screening suitable pathways [14].

### 2.7 Immune Checkpoint Expression

Using data from previous studies, we extracted information on immune checkpoints and analysed their expression levels in both HR and LR groups. The analysis was conducted using the “reshape2” package for data manipulation and processing [15].

### 2.8 Tumour Mutational Burden

Tumour somatic mutation data were collected from the TCGA and subsequently processed using the “TCGA biolinks” package. Waterfall plots were created in R utilising the “maftools” package, and the TMB was calculated [16, 17].

### 2.9 Evaluation of the TME and Immune Cell Distribution

To assess differences in stromal score, ESTIMATE score, immune score and tumour purity between the HR and LR groups, we utilised the “ESTIMATE” R package. Immune‐related data were further downloaded from the Tumour Immune Estimation Resource (TIMER) 2.0 website. A variety of computational tools were employed to refine the connection between risk scores and specific immune cell types [18]. For a more detailed analysis of immune cell composition, we performed immune infiltration analysis on 22 immune cell types. Furthermore, we used the single‐sample gene set enrichment analysis (ssGSEA) to compute function scores and compare these scores between the HR and LR groups.

### 2.10 Chemotherapy Assessment of TNF‐Related Signature

To explore tumour immune dysfunction and rejection (TIDE) in KIRC patients across both groups, we obtained TIDE scores and associated information from the TIDE website (https://tide.dfci.harvard.edu/) [19]. Furthermore, we calculated half‐maximal inhibitory concentrations (IC50) for standard chemotherapeutic drugs with the “oncoPredict” R package [20]. To evaluate differences in IC50 values among different groups, we employed the Wilcoxon signed‐rank test. Lastly, we compared drug responses between HR and LR groups based on the IC50 values.

### 2.11 Validation by Quantitative Reverse Transcription Polymerase Chain Reaction (RT‐qPCR) Three Tumour Cell Lines and One Normal Cell

KIRC cell lines, including 786‐O, A‐498, ACHN and the normal HK‐2 cell line, were obtained from Procell Life Science & Technology Co. Ltd. (Wuhan, China). RNA extraction was carried out using TRIzol reagent (Invitrogen, USA) and an RNA extraction kit. The RNA was reverse transcribed into cDNA using the PrimeScript RT Reagent Kit (Takara, Japan). TRmRNA expression levels, as detailed in Table S1, were quantified by RT‐qPCR, with relative RNA levels calculated using the 2^−ΔΔCT^ method [21].

### 2.12 Construct an External Validation Model of TRmRNAs

The E‐MTAB‐1980 data was retrieved from the ArrayExpress database, and we created ROC curves integrating clinical factors. Survival analyses were then conducted across patient subgroups to assess the prognosis of patients. Independent prognostic factors were identified through u/mCox. To estimate OS at 1, 3 and 5 years for KIRC, we utilised the “rms”, “timeROC” and “tidyverse” R packages, ensuring robust survival predictions [22].

## 3 Results

### 3.1 Screening for TRmRNAs in KIRC Patients

**FIGURE 1:**
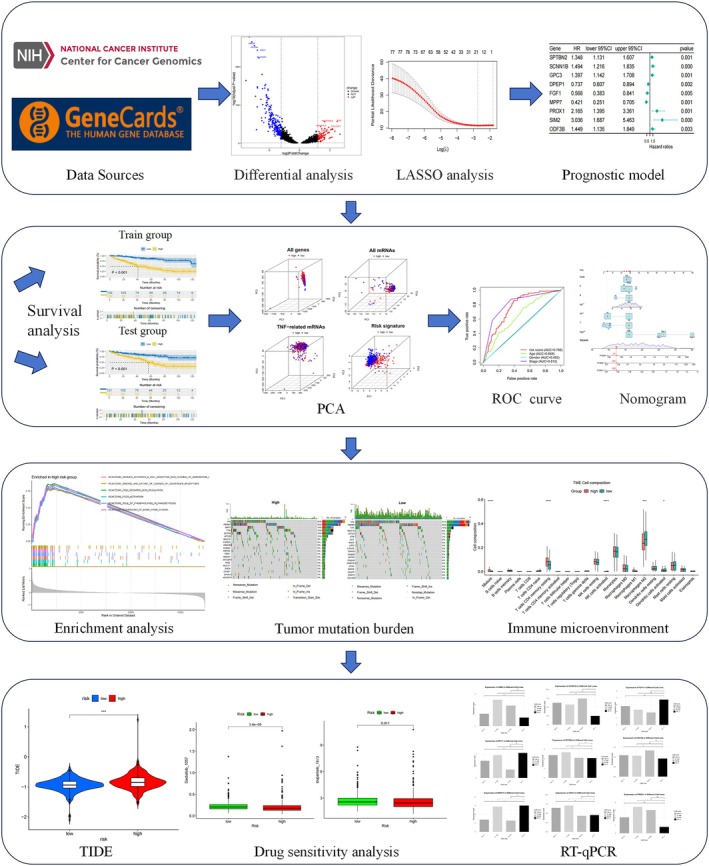
Flow Chart.

**FIGURE 2:**
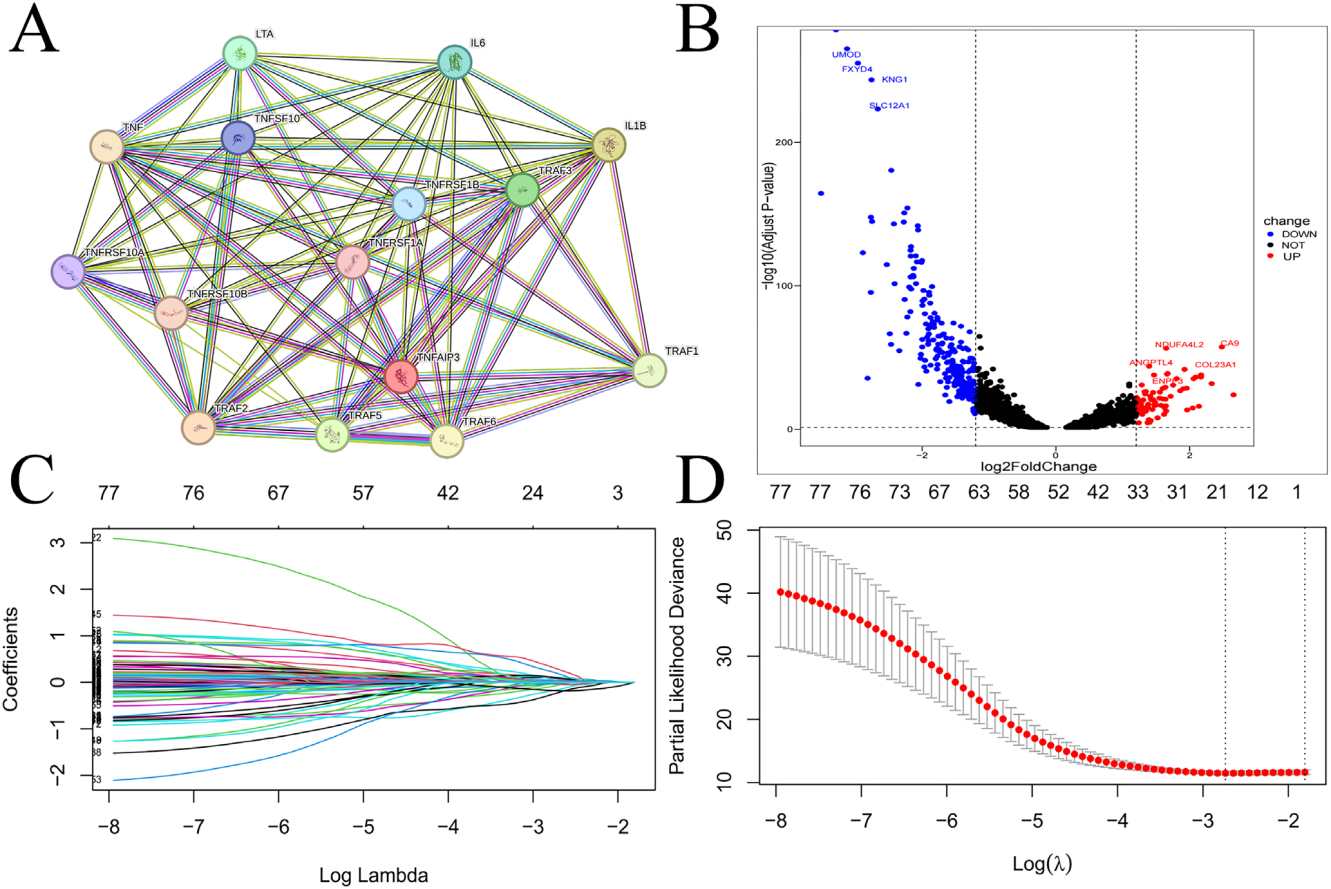
PPI network (A); Filter for TNF‐related mRNAs. Volcano plot of differentially expressed TNF‐associated mRNAs (B); LASSO regression analysis (C, D).

The rationale for our study is presented in a flowchart (Figure 1). First, we selected TRGs and developed protein–protein interaction (PPI) networks for 15 of these genes via the STRING database (https://string‐db.org/) (Figure 2A). Next, Pearson correlation analysis was conducted on TRGs, revealing significant associations between the expression of 17,846 mRNAs and TRGs (|Pearson R| > 0.3, *p* < 0.05). After refining these to 341 mRNAs, a differential expression analysis was performed, highlighting a significant difference in tumour tissue expression (Figure 2B).

Following the differential expression analysis, we randomly assigned 526 patients to test and train groups (Table 1). The uCox regression signature was employed to identify 78 mRNAs in the train cohort that were significantly related to differential prognosis (*p* < 0.05) (Table S2). Next, LASSO analysis was conducted, which led to the identification of 20 TRmRNAs linked to KIRC (Figure 2C,D) (Table S3). Subsequently, mCox regression analysis further reduced the list to 9 mRNAs that were significantly correlated with OS (Figure S1A). The interrelationships between these 9 mRNAs were explored (Figure S1B), along with their associations with TRGs (Figure S1C). Importantly, these mRNAs showed significant upregulation and downregulation in tumour tissues (Figure S1D).

**TABLE 1:** Clinical information of the patients in the testing and training groups.

Characteristics	Train cohort (*n* = 264)		Test cohort (*n* = 262)		Entire cohort (*n* = 526)	
	*n*	%	*n*	%	*n*	%
Age						
≤ 65	173	65.53	174	66.41	347	65.97
> 65	91	34.47	88	33.59	179	34.03
Status						
Alive	178	67.42	177	67.56	355	67.49
Dead	86	32.58	85	32.44	171	32.51
Gender						
Female	94	35.61	89	33.97	183	34.79
Male	170	64.39	173	66.03	343	65.21
Stage						
Stage I	146	55.30	115	43.89	261	49.62
Stage II	22	8.33	35	13.36	57	10.84
Stage III	53	20.08	70	26.71	123	23.38
Stage IV	42	15.91	40	15.27	82	15.59
Unknown	1	0.38	2	0.76	3	0.57
T stage						
T1	150	56.82	117	44.66	267	50.76
T2	28	10.61	41	15.65	69	13.12
T3	81	30.68	98	37.40	179	34.03
T4	5	1.89	6	2.29	11	2.09
M stage						
M0	205	77.65	213	81.30	418	79.47
M1	58	21.97	38	14.50	96	18.25
Unknown	1	0.38	11	4.20	12	2.28
N stage						
N0	102	38.64	136	51.91	238	45.25
N1	9	3.41	7	2.67	16	3.04
Unknown	153	57.95	119	45.42	272	51.71
Race						
White	226	85.61	231	88.17	457	86.88
Black or African American	29	10.98	25	9.54	54	10.27
Asian	5	1.89	3	1.15	8	1.52
Unknown	4	1.52	3	1.15	7	1.33

Abbreviations: M stage, metastasis stage; N stage, node stage; T stage, tumour stage.

### 3.2 Development and Verification of the Predictive Model

**FIGURE 3:**
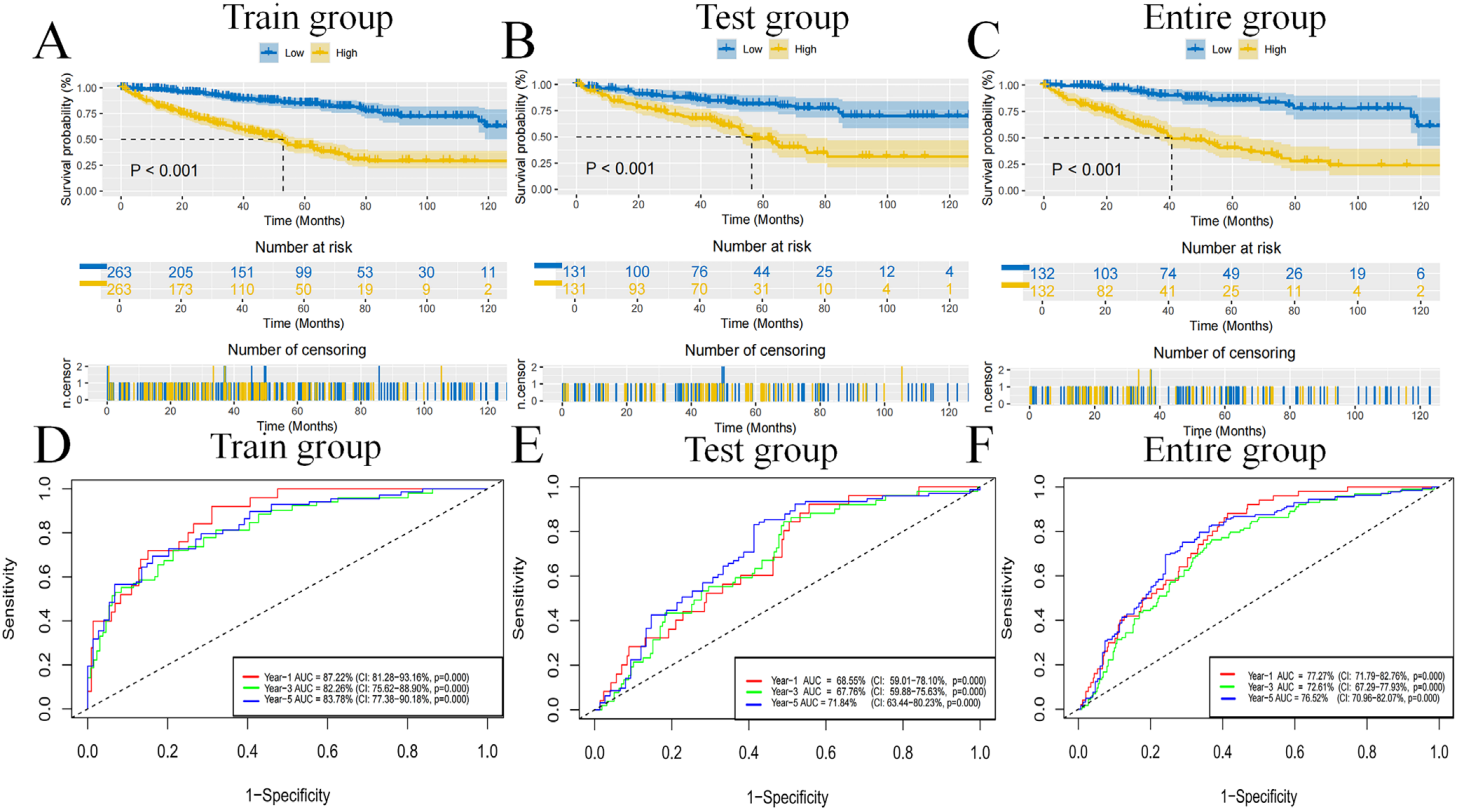
The model prediction effect is validated by the train group, test group, and entire group. K‐M analysis (A–C) and time‐dependent ROC curves (D–F) to compare the survival of the high‐risk group and low‐risk group.

Using the nine TRmRNAs, we established a formula to calculate risk scores for each patient: Risk score = coefficient (SPTBN2) × expression (SPTBN2) + coefficient (SCNN1B) × expression (SCNN1B) + coefficient (GPC3) × expression (GPC3) + coefficient (DPEP1) × expression (DPEP1) + coefficient (FGF1) × expression (FGF1) + coefficient (MPP7) × expression (MPP7) + coefficient (PROX1) × expression (PROX1) + coefficient (SIM2) × expression (SIM2) + coefficient (ODF3B) × expression (ODF3B). Table 2 provides an overview of clinical characteristics for individuals in both HR and LR groups. In datasets for train, test and the total population, the HR group showed significantly poorer survival than the LR group (*p* < 0.001) (Figure 3A–C). The time‐dependent ROC curves verified strong model predictions, with Area Under the Curve (AUC) of 0.872 (95% CI: 0.813–0.932, *p* < 0.001), 0.823 (95% CI: 0.756–0.889, *p* < 0.001) and 0.838 (95% CI: 0.774–0.902, *p* < 0.001) for one, three and five years in the training group; 0.686 (95% CI: 0.590–0.781, *p* < 0.001), 0.678 (95% CI: 0.599–0.756, *p* < 0.001) and 0.718 (95% CI: 0.634–0.802, *p* < 0.001) in the test group; and 0.773 (95% CI: 0.718–0.828, *p* < 0.001), 0.726 (95% CI: 0.673–0.779, *p* < 0.001) and 0.765 (95% CI: 0.710–0.821, *p* < 0.001) in the overall group (Figure 3D–F). Additional visualisations, including the expression profiles of the 9 mRNAs in patients with different risk levels (Figure S2A–C), risk curves (Figure S2D–F), risk distribution plots (Figure S2G–I) and scatter plots (Figure S2J–L), all support the model's prognostic value.

**TABLE 2:** Clinical information for 526 patients in different risk categories.

Characteristics	High‐risk group (*n* = 263)		Low‐risk group (*n* = 263)	
	*n*	%	*n*	%
Age				
≤ 65	166	63.12	181	68.82
> 65	97	36.88	82	31.18
Status				
Alive	132	50.95	223	84.79
Dead	131	49.05	40	15.21
Gender				
Female	94	35.74	89	33.84
Male	169	64.26	174	66.16
Stage				
Stage I	93	35.36	168	63.88
Stage II	33	12.55	24	9.13
Stage III	71	27.00	52	19.77
Stage IV	64	24.33	18	6.84
Unknown	2	0.76	1	0.38
T stage				
T1	98	37.26	169	64.26
T2	42	15.97	27	10.27
T3	112	42.59	67	25.48
T4	11	4.18	0	0
M stage				
M0	184	69.96	234	88.97
M1	60	22.81	18	6.84
Unknown	19	7.22	11	4.18
N stage				
N0	129	49.05	109	41.44
N1	12	4.56	4	1.52
Unknown	122	46.39	150	57.03
Race				
White	223	85.93	234	88.97
Black or African American	34	12.93	20	7.60
Asian	3	1.14	5	1.90
Unknown	3	1.14	4	1.52

Abbreviations: M stage, metastasis stage; N stage, node stage; T stage, tumour stage.

**FIGURE 4:**
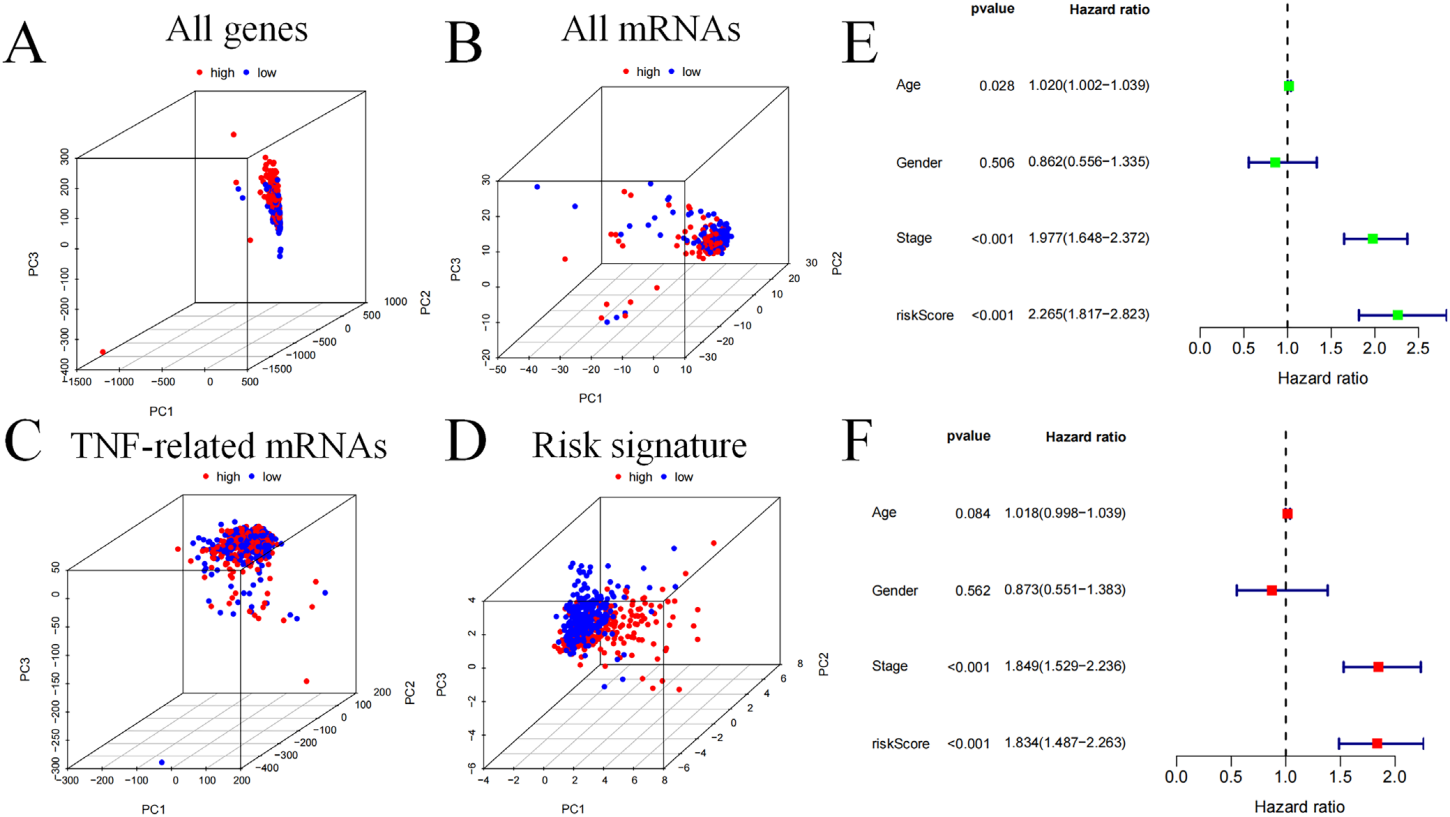
PCA and independent prognostic analysis of the signature. PCA based on all genes (A), all mRNAs (B), TNF‐related mRNAs (C), and risk signature (D); univariate (E) and multivariate (F) independent prognostic analysis.

PCA analysis (Figure 4A–D) revealed clear separation between HR and LR groups. Using uCox regression, age, tumour stage and risk score were determined to be standalone prognostic variables (HR = 1.02, 1.98 and 2.27; 95% CI amount = 1.00–1.04, 1.65–2.37 and 1.82–2.82; *p* < 0.05, *p* < 0.001 and *p* < 0.001) (Figure 4E). Moreover, mCox regression validated that the stage and risk score continued to be significant independent factors of OS (HR = 1.85 and 1.83; 95% CI amount = 1.53–2.24 and 1.49–2.26; Both P values < 0.001) (Figure 4F).

**FIGURE 5:**
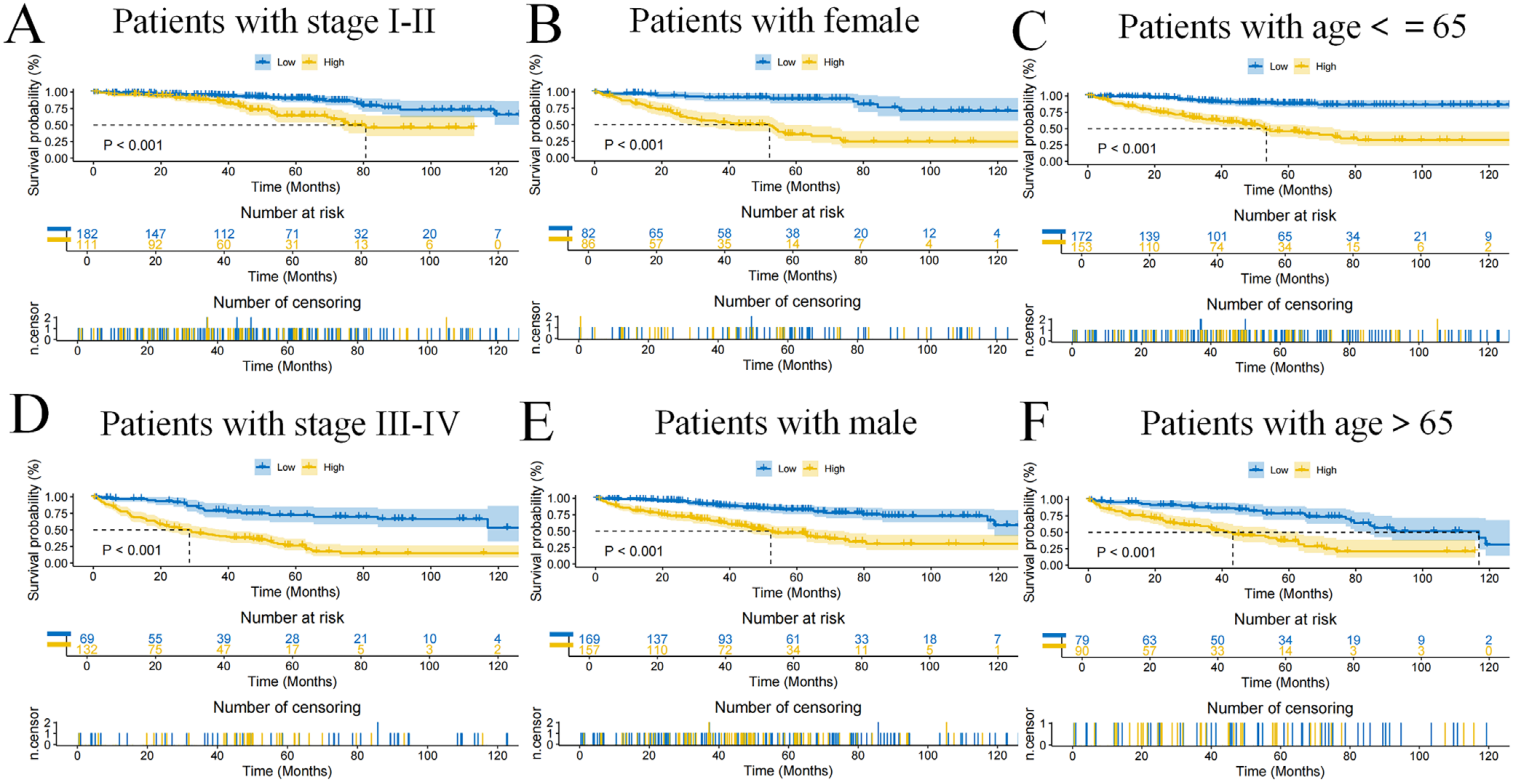
Further validation of model effects. Survival curves of patients in different clinical states (A–F).

The heatmap, which incorporates various clinical features, risk groups and mRNAs associated with the prognostic model (Figure S3A), along with survival curves for patients across different clinical subgroups (Figure 5), demonstrates the robust predictive power of the model. Significant differences in survival were observed among various clinical categories. To confirm the robustness of our model and exclude the possibility that its performance arose by random chance, we conducted a permutation test with 1000 iterations. The results revealed that the original model's C‐index and AUC values were significantly higher than those obtained from the permuted datasets (*p* < 0.001), suggesting that the predictive ability of the model is unlikely to be due to chance. A visual comparison of the performance distributions is provided in Figure S11.

### 3.3 Nomogram for Predicting Clinical OS

**FIGURE 6:**
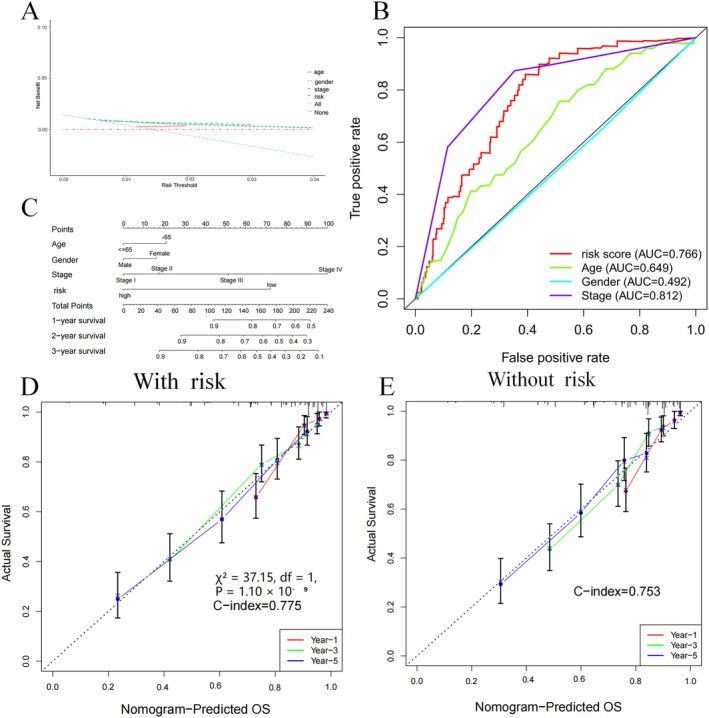
Nomogram predicts patient prognosis. Decision curve to test for forecast value (A) ROC curves containing different clinical information (B) A clinical prognosis nomogram is constructed by age, gender, risk, and stage together (C). Nomogram with (D) and without (E) risk model.

Evaluation of the link between risk score and clinical factors indicated that age (*p* = 0.51) showed no significant relationship with the risk score, whereas clinical stage (*p* < 0.001) exhibited a strong link (Figure S3B,C). Decision curve analysis (DCA) and ROC curve analysis (Figure 6A,B) showed that the risk score offered superior predictive value compared to other clinical factors. A prospective estimator for KIRC patients, which integrated clinical factors, was developed (Figure 6C); the AUC for the risk score in predicting 3‐year overall survival (OS) was 0.766, which was comparable to TNM stage (AUC = 0.812) and superior to age (AUC = 0.649) and sex (AUC = 0.492), indicating that the risk score provides robust prognostic value, and its validation using patient data yielded promising results (Figure S4). To formally assess the additive prognostic value of the nomogram, we compared the combined model (TNM stage + TRmRNA‐based risk score) with a model based solely on the TNM stage. The likelihood ratio test confirmed that the addition of the risk score significantly improved model fit (*χ* ^2^ = 37.15, df = 1, *p* = 1.10 × 10^−9^), suggesting that the TRmRNA risk score provides meaningful prognostic information beyond conventional clinical staging (Figure 6D,E).

### 3.4 Enrichment Analysis

The Kyoto Encyclopedia of Genes and Genomes (KEGG) enrichment analysis showed functions of model‐related genes, such as neuroactive ligand–receptor interaction, cell adhesion molecules, collecting duct acid secretion, viral protein interaction with cytokines and cytokine receptors, cytokine–cytokine receptor interaction, protein digestion and absorption and phagosome formation (Figure S5). GSEA further revealed distinct pathway enrichments across various gene sets. In the HR group, the GOBP library showed significant enrichment in functions related to immunoglobulin production, B cell‐mediated immunity, lymphocyte‐mediated immunity, molecular mediator production for immune response, immunoglobulin complex formation and antigen binding (Figure S6). A detailed list of the enriched pathways can be found in Table S4.

### 3.5 Tumour Mutation Burden

**FIGURE 7:**
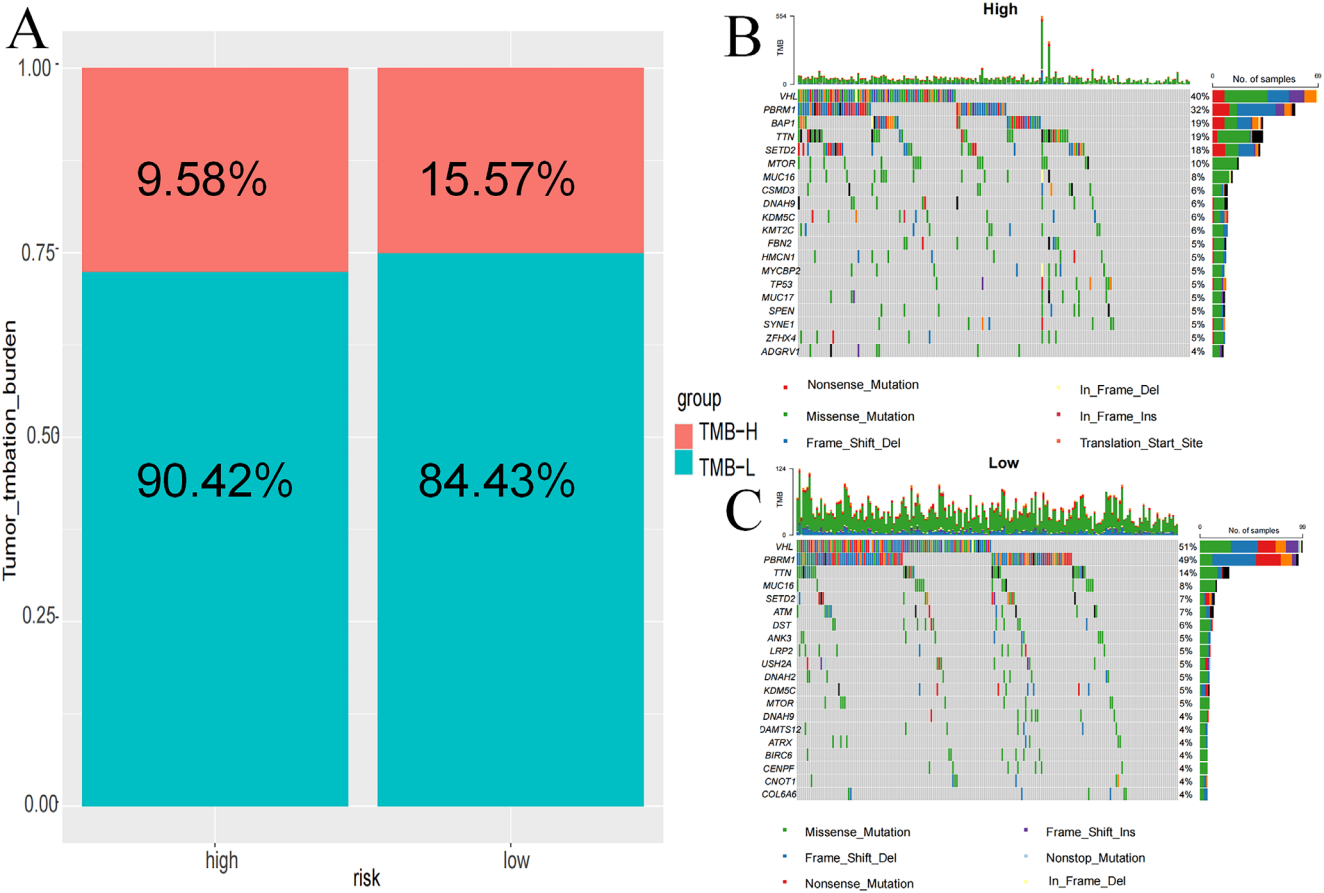
Tumour mutation burden in different risk groups. Percentage bar graph showing TMB for different risk subgroups (A) High‐risk group waterfall chart (B) Low‐risk group waterfall chart (C).

TMB was evaluated in patients, revealing a significantly higher TMB in the HR (Figure 7A). To further investigate the genetic landscape, a waterfall plot visualised the 20 most mutated genes across subgroups (Figure 7B,C). Among the HR group, the most commonly mutated genes were VHL (40%), PBRM1 (32%), BAP1 (19%), TTN (19%) and SETD2 (18%). In contrast, LR patients showed the highest mutation frequencies in VHL (51%), PBRM1 (49%), TTN (14%), SETD2 (7%) and MUC16 (8%).

### 3.6 Tumour Immune Infiltration Status Analysis

**FIGURE 8:**
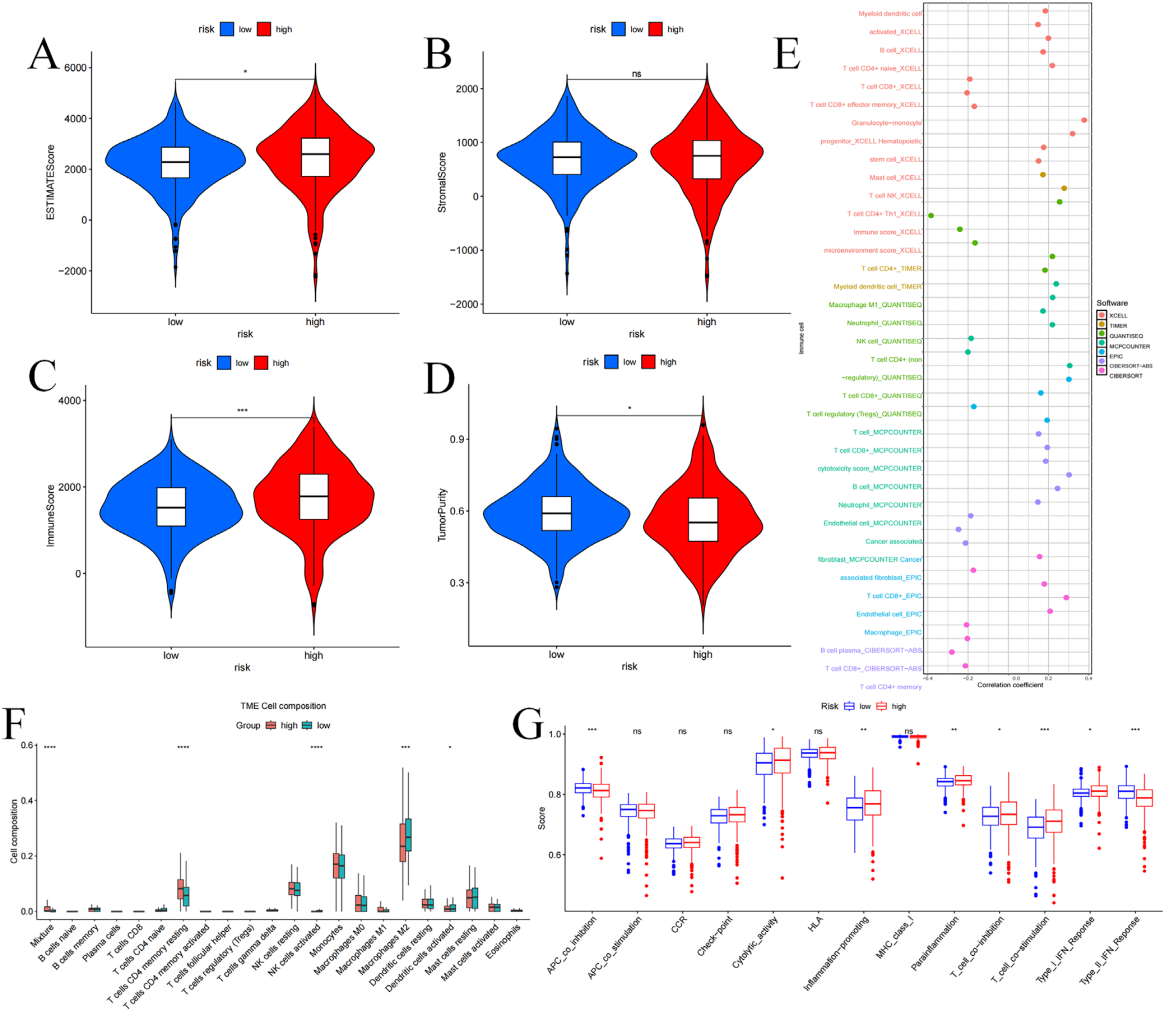
Analysis of tumour immune microenvironment. Violin plots of differences in ESTIMATE scores (A), stromal scores (B), immune scores (C) and tumour purity (D) for different risk subgroups; Bubble plots of correlations between immune cells and risk scores under six algorithms (E); Proportions of 22 immune cells in two subgroups under the CIBERSORT algorithm (F); single sample gene set enrichment analysis (G). *p < 0.05, **p < 0.01, ***p < 0.001.

TME analysis identified elevated ESTIMATE scores (*p* < 0.05; Figure 8A) and Immune scores (*p* < 0.001; Figure 8C) in the HR group, while the Stromal score showed no significant variation (*p* > 0.05; Figure 8B). Also, the HR group displayed reduced tumour purity (*p* < 0.05; Figure 8D). These observations highlight that HR is linked to an immune‐suppressive tumour microenvironment, potentially impairing the antitumor immune response. We also examined the connection between immune cell composition and risk scores using multiple algorithms (Figure 8E). Figure 8F exhibited that in the HR group, there is a higher proportion of T cells CD4 memory resting (*p* < 0.001). In contrast, the LR group showed an increased proportion of NK cells activated (*p* < 0.001) and Macrophages M2 (*p* < 0.001). The connection between immune cells and risk scores is illustrated (Figure S7). Functional pathway analysis revealed a reduction in type II IFN response (*p* < 0.001) and APC coinhibition (*p* < 0.001) in HR patients, whereas pathways such as T cell stimulation (*p* < 0.001) and type I IFN response (*p* < 0.05) were upregulated (Figure 8G).

### 3.7 Benefits of Predictive Models for KIRC Treatment

TIDE revealed that both T cell exclusion and T cell dysfunction were significantly more pronounced in the HR group with TNF‐associated signatures (Figure S8A–C). This suggests that the HR subgroup has a higher potential for immune evasion, which could complicate therapeutic interventions. Further research identified 19 differentially expressed immune checkpoints including PPP6R1, IL15RA and DDX47 that were upregulated in the HR group (Figure S8D), which indicates that the HR group may benefit from immune therapies targeting these specific immune checkpoints. Additionally, we assessed the sensitivity of tumours in both the LR and HR groups to various anti‐tumour drugs. Drugs exhibiting differential efficacy between the subgroups are listed in Table S5 (LR) and S6 (HR), while those with no notable differences are shown in Table S7. The HR patients notably revealed heightened sensitivity to therapies aimed at the PI3K/MTOR signalling pathway, which may guide the decision of targeted therapies.

### 3.8 Risk Model Validation In Vitro

Immunohistochemical images, obtained from the HPA website (Figure S9A), illustrated the differences in protein expression of TRGs in KIRC and nearby normal tissues. RT‐qPCR analysis demonstrated that, of nine TRmRNAs, DPEP1, FGF1 and MPP7 were largely expressed in normal tissues, whereas the other six mRNAs exhibited elevated expression levels in tumour tissues (Figure S9B).

### 3.9 Validation of the Prognostic Traits Externally

To assess the predictive value of the signature, we developed an external validation using the E‐MTAB‐1980 cohort [23]. K‐M analysis found that patients in the HR group experienced worse outcomes than those in the LR group (*p* = 0.003; Figure S10A). The model's robustness was further confirmed through ROC curve analysis, which produced AUC of 0.871, 0.776 and 0.732 for 1, 3 and 5 years (Figure S10B). Importantly, the AUC for the risk score (0.896) was notably higher than those for age (0.573) and gender (0.627), highlighting the excellent prognostic capability of the risk score model (Figure S10C). To validate the TRmRNA signature's predictive value across various clinical subgroups, uCox regression models were performed, with stage (HR = 2.28, 95% CI amount 1.68–3.10, *p* < 0.001) and risk score (HR = 2.17, 95% CI amount 1.66–2.84, *p* < 0.001) (Figure S10D). The mCox analysis had similar results: stage (HR = 2.03, 95% CI amount 1.42–2.90, *p* < 0.001) and risk score (HR = 1.80, 95% CI amount 1.31–2.45, *p* < 0.001) (Figure S10E). The heatmap demonstrated a clear survival difference between the LR and HR groups, with the LR group showing significantly longer OS (Figure S10F). Despite potential cross‐platform variability between RNA‐seq (TCGA) and microarray (E‐MTAB‐1980), our model exhibited consistently high prognostic performance, suggesting that the core biological signals captured by TRmRNAs are stable and generalisable across platforms.

## 4 Discussion

Over the past few years, KIRC incidence has steadily increased, posing significant challenges in prognosis prediction and therapeutic decision‐making. Despite advancements in surgical and systemic treatments, the heterogeneity in patient outcomes highlights the limitations of current staging systems, such as the AJCC TNM classification, which fail to capture the molecular complexity of tumours. In response to these challenges, biomarker‐based prognostic models have emerged as promising tools to enhance prediction accuracy [3]. While TNF‐related gene signatures have been applied in malignancies such as cervical squamous cell carcinoma and bladder cancer, their applicability to kidney renal clear cell carcinoma (KIRC) remains limited [24]. KIRC exhibits distinct biological and immunological hallmarks—particularly immune evasion and pronounced vascularisation—that necessitate cancer‐specific prognostic modelling. In our research, we developed and validated a predictive signature utilising nine TRmRNAs, which demonstrated extremely high accuracy in assessing prognosis in HR and LR groups. Additionally, we integrated this molecular model with clinical factors into a nomogram, providing a more comprehensive approach for predicting survival outcomes. By incorporating enrichment analysis, TMB evaluation and drug sensitivity predictions, our study provides important perspectives for the development of personalised treatment strategies in KIRC.

In this study, we propose a novel prognostic model for kidney renal clear cell carcinoma (KIRC) based on a refined subset of TNF family‐related mRNAs. Unlike previous models—including those by Huang et al. and Zhang et al. [25, 26]. That employed broad TNF gene panels in lung adenocarcinoma—our approach tailors gene selection to the distinct immune and molecular landscape of KIRC. This cancer‐specific focus enhances both prognostic precision and clinical relevance. This model exhibited high predictive accuracy across multiple evaluation metrics, including the ROC curve, C‐index and calibration curve. Notably, our risk model demonstrated prognostic power comparable to that of the TNM staging system and outperformed age and sex in ROC analysis. When integrated into a nomogram with standard clinical variables, the inclusion of the TRmRNA risk score significantly improved predictive accuracy, as reflected by a higher C‐index. These results notably surpassed the performance of the TNF‐related model proposed by Ma et al. [27]. Our approach, utilising TNF‐related genes to construct prognostic signatures, aligns with similar studies in cervical and bladder cancers, further validating the robustness of this method [28]. Several key mRNAs in our model, such as PROX1 and ODF3B, are well‐established in tumour biology, further reinforcing the biological relevance of our prediction framework. Specifically, Lv et al. have shown that PROX1 promotes KIRC cell proliferation [29]. Also, ODF3B, a gene closely linked to glioma growth and apoptosis, has been reported to exhibit elevated expression levels in gliomas. Notably, higher expression of ODF3B is correlated with a poorer prediction in glioma patients [30]. **SCNN1B** is downregulated in KIRC due to promoter hypermethylation and may preserve epithelial integrity [31]. **GPC3** and **MPP7** inhibit proliferation and invasion through modulation of growth factor signalling and maintenance of epithelial polarity, respectively [32, 33]. **DPEP1** is involved in immune‐metabolic regulation, and its low expression is linked to poor prognosis. Conversely, some TRmRNAs appear oncogenic [34]. **FGF1** promotes RCC proliferation via PI3K/Akt and MAPK pathways [35]. **SIM2** is linked to increased invasion and Wnt pathway activation [29, 36]. Additionally, DCA indicated that our risk score outperforms traditional prognostic predictors, offering a superior net benefit in risk assessment [37]. Together, the TNF‐related prediction model demonstrates potential as an effective and dependable approach to enhance prognostic assessment for cancer patients.

To further investigate the prognostic differences and molecular alterations between HR and LR groups, we conducted pathway enrichment and immune function analysis in the tumour microenvironment using our predictive model. Functional enrichment analysis showed that the HR group displayed increased expression of pathways related to immunity, indicating an activated but dysregulated immune response [38]. Among these pathways, the enrichment of CD22‐mediated BCR regulation in the high‐risk group may represent an immunosuppressive mechanism mediated by dysfunctional or regulatory B‐cell populations. CD22 negatively regulates BCR signalling and contributes to immune tolerance. Its increased activity in high‐risk tumours could suppress antigen presentation and cytotoxic immune responses, supporting immune escape and tumour progression [39]. This immune dysregulation may contribute to the more aggressive tumour behaviour observed in the HR group. Conversely, the LR group showed enrichment in solute carrier and proximal tubule transport pathways; these pathways are characteristic of functional proximal tubular epithelial cells and suggest that tumours in this group may retain more segment‐specific functions. The preservation of such renal epithelial programs may reflect a less aggressive tumour biology and explain the improved outcomes observed in this subgroup [40]. In addition, immune function analysis detected notable differences between the two risk groups in processes like antigen‐presenting cell (APC) co‐suppression, parainflammatory responses and type II interferon signalling. Notably, IFN‐γ has emerged as a pivotal mediator in the immune microenvironment of tumours. It is identified as a significant marker of immunotherapy effectiveness and a key factor in regulating tumour progression [41]. The high TMB observed in the HR group further supports the potential for immunotherapy, as TMB is typically associated with an increased burden of neoantigens. However, it also correlates with poorer prognosis in KIRC patients [42, 43]. Additionally, the inactivation of tumour suppressor genes such as VHL has been implicated in cancer progression. Loss of VHL function facilitates histone lactylation, which activates the expression of platelet‐derived growth factor receptor β (PDGFRβ) at the transcriptional level, a key factor in KIRC development [44]. Overall, the poorer prognosis observed in the HR group is closely linked to elevated TMB, dysregulated immune‐related pathways and inactivation of tumour suppressor genes. These findings underscore important mechanisms driving KIRC progression and provide important guidance for identifying potential therapeutic targets and research directions.

As previously discussed, TMB is a critical determinant of immunotherapy efficacy in cancer treatment. Studies suggest that patients with high TMB are prone to respond well to immunotherapy [45]. In our study, the HR group exhibited elevated TMB levels, indicating that these patients may be more responsive to immunotherapeutic interventions. To further investigate the potential for personalised treatment approaches, we compared the immunotherapy responses between the HR and LR groups; although these drug sensitivity predictions were based on in silico IC50 estimation from transcriptomic data, rather than real‐world clinical responses, they provide a preliminary pharmacogenomic framework that warrants further experimental and clinical validation. Notably, HR patients exhibited heightened responsiveness to therapies aimed at the PI3K/MTOR signalling pathway, such as Dactolisib and Buparlisib, consistent with the drug sensitivity findings, suggesting elevated pathway activity that may contribute to both poor prognosis and enhanced sensitivity to pathway‐specific inhibitors. Notably, Buparlisib has been shown to downregulate epithelial‐mesenchymal transition pathways and matrix metalloproteinases, thereby limiting metastasis in urinary tract cancers, indicating promising therapeutic potential [46, 47]. These findings align with the recognised role of PI3K/MTOR signalling in developing tumour growth and conferring resistance to conventional therapies [48, 49], likely due to aberrant activation of this pathway. Ye et al. demonstrated that GNE‐477 effectively suppressed RCC growth by blocking PI3K/AKT/mTOR signalling [50]. The enrichment of pro‐survival and immunosuppressive features in high‐risk patients suggests greater pathway dependency, supporting their potential responsiveness to targeted inhibition. In contrast, LR patients demonstrated higher responsiveness to agents such as Cediranib and Osimertinib, which specifically target the VEGFR and EGFR pathways, respectively [51]. This variation in drug sensitivity between the two groups reflects distinct molecular and immune characteristics associated with each risk profile. Additionally, the elevated expression of immune checkpoints, including IL15RA and DDX47, in the HR group suggests that these individuals might benefit from immune checkpoint inhibitors (ICIs). DDX47, an RNA‐binding protein, has been closely linked to KIRC progression [52, 53]. Recent studies have highlighted the effectiveness of ICIs in treating KIRC, particularly in patients with high TMB or increased expression of immune checkpoints [54, 55]. These observations emphasise the potential of integrating risk‐based stratification with immunotherapy strategies, which could significantly improve therapeutic outcomes for KIRC patients.

Our study makes several key contributions to the field of KIRC research. Firstly, our bioinformatics‐based pipeline is in line with recent prognostic studies in oncology, such as those reported by Dang et al. and Tran et al., supporting the methodological soundness and translational potential of our approach [56, 57]. We present a prognostic model that demonstrates high predictive accuracy, surpassing similar models, thus establishing TRmRNAs as promising biomarkers for KIRC prognosis. Secondly, we strengthen the validity of our findings by performing external validation using independent datasets, which enhances both the biological relevance and reliability of our results [58]. While the final model includes only nine TRmRNAs, we acknowledge the potential risk of overfitting due to the limited sample size. To reduce this risk, we applied stringent feature selection (univariate Cox, LASSO and ML‐based filtering), and more importantly, validated the model in an external cohort (E‐MTAB‐1980), where it achieved robust predictive performance. These results support that the model captures reproducible prognostic signals rather than overfitting noise and demonstrate its generalisability across independent datasets. Thirdly, our study is the first to investigate the link between TRmRNAs and KIRC prognosis, highlighting these mRNAs as potential biomarkers for the disease. Although our TRmRNA‐based prognostic model was validated in two independent public datasets, the lack of prospective clinical validation remains a key limitation. As this study is retrospective in nature, future work will involve prospective, multicentre cohorts to evaluate the model's clinical utility in risk stratification and treatment guidance. While no direct functional experiments were performed, our results provide a hypothesis‐generating framework. Follow‐up studies will include genetic perturbation and phenotypic assays in cellular and animal models to elucidate the biological roles of key TRmRNAs. Notably, external validation using the E‐MTAB‐1980 dataset supports the model's robustness. Nevertheless, residual batch effects cannot be fully excluded, and overfitting from random train‐test splits may still occur. Although feature selection and model training were strictly limited to the training cohort, future validation strategies will consider cohort‐level or subtype‐aware designs to minimise data leakage. In conclusion, our model shows strong prognostic potential, but further experimental and clinical validation is essential to confirm its applicability in KIRC.

## 5 Conclusion

Our risk model, developed based on 9 TRmRNAs, demonstrates strong predictive accuracy for patient outcomes. When integrated with clinical factors, the accompanying nomogram further enhances its predictive power, offering exceptional effectiveness in forecasting survival. The poor prognosis observed in HR patients is strongly correlated with factors such as elevated TMB, enrichment of CD22‐mediated BCR regulation and immunoglobulin complex pathways, etc. The results of drug susceptibility analysis provide valuable information that can help in the creation of personalised treatments. However, additional clinical trial validation is essential to fully realise its applicability and impact in clinical practice.

## Author Contributions

**Zijian Hu:** conceptualization (equal), data curation (equal), formal analysis (equal), investigation (equal), methodology (equal), project administration (equal), resources (equal), software (equal), supervision (equal), validation (equal), visualization (equal), writing – original draft (lead), writing – review and editing (equal). **Yajie Zhou:** conceptualization (equal), data curation (equal), formal analysis (equal), investigation (equal). **Lei Xie:** conceptualization (equal), data curation (equal), formal analysis (equal), investigation (equal), methodology (equal). **Shuwen Zhang:** conceptualization (equal), data curation (equal), formal analysis (equal), investigation (equal), methodology (equal), writing – original draft (equal). **Yijiang Liu:** conceptualization (equal), data curation (equal), formal analysis (equal), investigation (equal), methodology (equal), writing – original draft (equal). **Wenxiong Zhang:** conceptualization (equal), data curation (equal), formal analysis (equal), funding acquisition (lead), investigation (equal), methodology (equal), project administration (equal), resources (equal), software (equal), supervision (equal), validation (equal), visualization (equal), writing – original draft (equal), writing – review and editing (lead). **Ting Huang:** conceptualization (equal), data curation (equal), formal analysis (equal), investigation (equal), methodology (equal), project administration (equal), resources (equal), software (equal), supervision (equal), validation (equal), visualization (equal), writing – original draft (equal), writing – review and editing (equal).

## Ethics Statement

This article does not contain any studies with human participants or animals performed by any of the authors.

## Consent

For this type of study formal consent is not required.

## Conflicts of Interest

The authors declare no conflicts of interest.

## Supporting information


**Figure S1.** Construct a prognostic model. Forest plots associated with multifactor regression (A); Correlation circle plot of 9 TNF‐related mRNAs (B) Mulberry plots of 9 TNF‐related mRNAs with TNF‐related genes (C) Deviation plots indicating upregulation and downregulation changes of 9 TNF‐related mRNAs (D).

**Figure S2.** The model prediction effect is validated by the train group, test group and the entire group. Heat map of 9 TNF‐related mRNAs expressions (A‐C). Risk curve for risk scores (D‐F) and Scatterplot (G‐I) for the survival status of each patient; Scatterplot of risk scores of patients with different survival statuses (J‐L).

**Figure S3.** Further validation of model effects. mRNA signature expression heat map with clinical information (A); Correlation analysis of risk signature with age (B) and stage (C).

**Figure S4.** Nomogram with patient verification.

**Figure S5.** KEGG enrichment analysis. Bar chart (A); Circle chart (B).

**Figure S6.** GSEA enrichment analysis. Enrichment pathways in entire group in different gene sets (A‐F).

**Figure S7.** Correlation plot of risk scores with immune cells. Scatter plot of the correlation between risk scores and cancer associated fibroblast MCPCOUNTER (A), cancer associated fibroblast EPIC (B), T cell NK (C), T cell regulatory (Tregs) (D), Neutrophil (E), T cell (F), Myeloid dendritic cell (G), T cell follicular helper (H), T cell CD4+ Th1 (I), Macrophage M1 (J), Mast cell activated (K), NK cell (L), Mast cell activated (M), T cell follicular helper (N).

**Figure S8.** The TNF‐related mRNAs risk pattern in tumour therapy. Differences in dysfunction (A), immune exclusion (B), TIDE (C) and immune checkpoints (D) in high and low‐risk groups. * *p* < 0.05, ** *p* < 0.01, *** *p* < 0.001.

**Figure S9.** In vitro experimental validation of the risk model. Immunohistochemical staining images of partial TNF‐related gene proteins in KIRC tissue and normal tissue (A); Relative expression of 9 TNF‐related mRNAs in different risk subgroups (B). * *p* < 0.05, ** *p* < 0.01, *** *p* < 0.001, **** *p* < 0.0001.

**Figure S10.** External validation of prognostic characterisation. K‐M analysis (A) and Time‐dependent ROC curves (B) to compare the survival of the high‐risk group and low‐risk group. ROC curves containing different clinical information (C). Univariate (D) and multivariate (E) independent prognostic analysis. The heatmap between high‐risk and low‐risk group (F).

**Figure S11.** Results of permutation testing (1000 iterations). The histogram shows the distribution of C‐index values from permuted models. The red dashed line indicates the C‐index of the original model, which lies far in the tail of the null distribution (*p* < 0.001), confirming that the model’s performance is unlikely to occur by chance.

**Table S1.** Primer sequences for 9 TNF‐related mRNAs. mRNA signature expression heatmap (C).

**Table S2.** 78 differentially expressed TNF‐related mRNAs.

**Table S3.** 20 differentially expressed TNF‐related mRNAs.

**Table S4.** GSEA pathways for different risk groups.

**Table S5.** Antineoplastic drug sensitivity information (sensitive group: low).

**Table S6.** Antineoplastic drug sensitivity information (sensitive group: high).

**Table S7.** Antineoplastic drug sensitivity information (no obviously sensitive group).

## Data Availability

Data is provided within the manuscript or Supporting Information files.

## References

[1] R. L. Siegel , A. N. Giaquinto , and A. Jemal , “Cancer Statistics, 2024,” CA: A Cancer Journal for Clinicians 74, no. 1 (2024): 12–49. 38230766 10.3322/caac.21820

[2] R. J. Motzer , E. Jonasch , N. Agarwal , et al., “NCCN Guidelines Insights: Kidney Cancer, Version 2.2024,” Journal of the National Comprehensive Cancer Network 22, no. 1 (2024): 4–16. 38394781 10.6004/jnccn.2024.0008

[3] T. Klatte , S. H. Rossi , and G. D. Stewart , “Prognostic Factors and Prognostic Models for Renal Cell Carcinoma: A Literature Review,” World Journal of Urology 36, no. 12 (2018): 1943–1952. 29713755 10.1007/s00345-018-2309-4

[4] O. Ababneh , D. Nishizaki , S. Kato , and R. Kurzrock , “Tumor Necrosis Factor Superfamily Signaling: Life and Death in Cancer,” Cancer Metastasis Reviews 43, no. 4 (2024): 1137–1163. 39363128 10.1007/s10555-024-10206-6 PMC11554763

[5] J. H. Chen , X. Wu , Z. M. Wang , et al., “Tumor Necrosis Factor‐Related lncRNAs Predict Prognosis and Immunotherapy Response for Patients With Lung Adenocarcinoma,” Journal of Thoracic Disease 15, no. 3 (2023): 1373–1386. 37065578 10.21037/jtd-23-184 PMC10089852

[6] R. Yan , H. Zhu , P. Huang , et al., “Liquidambaric Acid Inhibits Wnt/β‐Catenin Signaling and Colon Cancer via Targeting TNF Receptor‐Associated Factor 2,” Cell Reports 38, no. 5 (2022): 110319. 35108540 10.1016/j.celrep.2022.110319

[7] D. Siegmund and H. Wajant , “TNF and TNF Receptors as Therapeutic Targets for Rheumatic Diseases and Beyond,” Nature Reviews Rheumatology 19, no. 9 (2023): 576–591. 37542139 10.1038/s41584-023-01002-7

[8] J. Zou , Z. Lin , W. Jiao , et al., “A Multi‐Omics‐Based Investigation of the Prognostic and Immunological Impact of Necroptosis‐Related mRNA in Patients With Cervical Squamous Carcinoma and Adenocarcinoma,” Scientific Reports 12, no. 1 (2022): 16773. 36202899 10.1038/s41598-022-20566-0 PMC9537508

[9] F. Jiang , H. Lin , H. Yan , X. Sun , J. Yang , and M. Dong , “Construction of mRNA Prognosis Signature Associated With Differentially Expressed Genes in Early Stage of Stomach Adenocarcinomas Based on TCGA and GEO Datasets,” European Journal of Medical Research 27, no. 1 (2022): 205. 36253873 10.1186/s40001-022-00827-4 PMC9578190

[10] M. J. Goldman , B. Craft , M. Hastie , et al., “Visualizing and Interpreting Cancer Genomics Data via the Xena Platform,” Nature Biotechnology 38, no. 6 (2020): 675–678. 10.1038/s41587-020-0546-8 PMC7386072 32444850

[11] C. Xu , Y. Li , W. Su , et al., “Identification of Immune Subtypes to Guide Immunotherapy and Targeted Therapy in Clear Cell Renal Cell Carcinoma,” Aging (Albany NY) 14, no. 17 (2022): 6917–6935. 36057262 10.18632/aging.204252 PMC9512512

[12] M. I. Love , W. Huber , and S. Anders , “Moderated Estimation of Fold Change and Dispersion for RNA‐Seq Data With DESeq2,” Genome Biology 15, no. 12 (2014): 550. 25516281 10.1186/s13059-014-0550-8 PMC4302049

[13] Q. Chen , S. Wang , and J. H. Lang , “Development and Validation of Nomogram With Tumor Microenvironment‐Related Genes and Clinical Factors for Predicting Overall Survival of Endometrial Cancer,” Journal of Cancer 12, no. 12 (2021): 3530–3538. 33995630 10.7150/jca.51493 PMC8120177

[14] A. Subramanian , P. Tamayo , V. K. Mootha , et al., “Gene Set Enrichment Analysis: A Knowledge‐Based Approach for Interpreting Genome‐Wide Expression Profiles,” Proceedings of the National Academy of Sciences of the United States of America 102, no. 43 (2005): 15545–15550. 16199517 10.1073/pnas.0506580102 PMC1239896

[15] M. Yi , T. Li , M. Niu , et al., “Exploiting Innate Immunity for Cancer Immunotherapy,” Molecular Cancer 22, no. 1 (2023): 187. 38008741 10.1186/s12943-023-01885-w PMC10680233

[16] A. Mayakonda , D. C. Lin , Y. Assenov , C. Plass , and H. P. Koeffler , “Maftools: Efficient and Comprehensive Analysis of Somatic Variants in Cancer,” Genome Research 28, no. 11 (2018): 1747–1756. 30341162 10.1101/gr.239244.118 PMC6211645

[17] A. Colaprico , T. C. Silva , C. Olsen , et al., “TCGAbiolinks: An R/Bioconductor Package for Integrative Analysis of TCGA Data,” Nucleic Acids Research 44, no. 8 (2016): e71. 26704973 10.1093/nar/gkv1507 PMC4856967

[18] T. Li , J. Fu , Z. Zeng , et al., “TIMER2.0 for Analysis of Tumor‐Infiltrating Immune Cells,” Nucleic Acids Research 48, no. W1 (2020): W509–W514. 32442275 10.1093/nar/gkaa407 PMC7319575

[19] P. Jiang , S. Gu , D. Pan , et al., “Signatures of T Cell Dysfunction and Exclusion Predict Cancer Immunotherapy Response,” Nature Medicine 24, no. 10 (2018): 1550–1558. 10.1038/s41591-018-0136-1 PMC6487502 30127393

[20] D. Maeser , R. F. Gruener , and R. S. Huang , “oncoPredict: An R Package for Predicting In Vivo or Cancer Patient Drug Response and Biomarkers From Cell Line Screening Data,” Briefings in Bioinformatics 22, no. 6 (2021): bbab260. 34260682 10.1093/bib/bbab260 PMC8574972

[21] W. Li , K. Ye , X. Li , et al., “YTHDC1 Is Downregulated by the YY1/HDAC2 Complex and Controls the Sensitivity of ccRCC to Sunitinib by Targeting the ANXA1‐MAPK Pathway,” Journal of Experimental & Clinical Cancer Research 41, no. 1 (2022): 250. 35974388 10.1186/s13046-022-02460-9 PMC9382764

[22] Y. Chen , Z. Wu , K. Cen , Y. Guo , and J. Jiang , “Development and Verification of a Novel Risk Model Related to Ubiquitination Linked With Prognosis and Therapeutic Response in Clear Cell Renal Cell Carcinoma,” Scientific Reports 14, no. 1 (2024): 25651. 39463392 10.1038/s41598-024-75948-3 PMC11514285

[23] S. Wang , T. Xiang , L. Yu , et al., “Novel Molecular Subtypes and Related Score Based on Histone Acetylation Modification in Renal Clear Cell Carcinoma,” Frontiers in Cell and Development Biology 9 (2021): 668810. 10.3389/fcell.2021.668810 PMC8495165 34631694

[24] F. Balkwill , “Tumour Necrosis Factor and Cancer,” Nature Reviews. Cancer 9, no. 5 (2009): 361–371. 19343034 10.1038/nrc2628

[25] H. Huang , H. Yu , X. Li , et al., “Genomic Analysis of TNF‐Related Genes With Prognosis and Characterization of the Tumor Immune Microenvironment in Lung Adenocarcinoma,” Frontiers in Immunology 13 (2022): 993890. 36505472 10.3389/fimmu.2022.993890 PMC9732939

[26] C. Zhang , G. Zhang , N. Sun , et al., “Comprehensive Molecular Analyses of a TNF Family‐Based Signature With Regard to Prognosis, Immune Features, and Biomarkers for Immunotherapy in Lung Adenocarcinoma,” eBioMedicine 59 (2020): 102959. 32853987 10.1016/j.ebiom.2020.102959 PMC7452643

[27] Y. Ma , X. Zhang , J. Yang , Y. Jin , Y. Xu , and J. Qiu , “Comprehensive Molecular Analyses of a TNF Family‐Based Gene Signature as a Potentially Novel Prognostic Biomarker for Cervical Cancer,” Frontiers in Oncology 12 (2022): 854615. 35392242 10.3389/fonc.2022.854615 PMC8980547

[28] H. Li , S. Liu , C. Li , Z. Xiao , J. Hu , and C. Zhao , “TNF Family‐Based Signature Predicts Prognosis, Tumor Microenvironment, and Molecular Subtypes in Bladder Carcinoma,” Frontiers in Cell and Development Biology 9 (2022): 800967. 10.3389/fcell.2021.800967 PMC8842074 35174161

[29] T. Lv , Y. Liu , J. Zhang , et al., “Impact of an Altered PROX1 Expression on Clinicopathology, Prognosis and Progression in Renal Cell Carcinoma,” PLoS One 9, no. 5 (2014): e95996. 24797520 10.1371/journal.pone.0095996 PMC4010401

[30] X. Z. Luan , S. X. Yuan , X. J. Chen , et al., “ODF3B Affects the Proliferation and Apoptosis of Glioma via the JAK/STAT Pathway,” American Journal of Cancer Research 14, no. 3 (2024): 1419–1432. 38590411 10.62347/GHKF1995 PMC10998755

[31] G. S. Dalgin , M. Drever , T. Williams , T. King , C. DeLisi , and L. S. Liou , “Identification of Novel Epigenetic Markers for Clear Cell Renal Cell Carcinoma,” Journal of Urology 180, no. 3 (2008): 1126–1130. 18639284 10.1016/j.juro.2008.04.137

[32] M. C. Valsechi , A. B. Oliveira , A. L. Conceição , et al., “GPC3 Reduces Cell Proliferation in Renal Carcinoma Cell Lines,” BMC Cancer 14 (2014): 631. 25168166 10.1186/1471-2407-14-631 PMC4161903

[33] L. Pei , X. Lv , G. Jia , X. Liang , X. Song , and A. Zhang , “CircSCNN1A Is a Tumor Suppressor in Renal Cell Carcinoma via Inducing the Upregulation of MPP7 by the Sponge Effect on miR‐421,” Transplant Immunology 75 (2022): 101736. 36343886 10.1016/j.trim.2022.101736

[34] Y. Zhang , Z. Chen , J. G. Chen , et al., “Ceruloplasmin Overexpression Is Associated With Oncogenic Pathways and Poorer Survival Rates in Clear‐Cell Renal Cell Carcinoma,” FEBS Open Bio 11, no. 11 (2021): 2988–3004. 10.1002/2211-5463.13283 PMC8564342 34449964

[35] U. Ramp , P. Reinecke , H. E. Gabbert , and C. D. Gerharz , “Differential Response to Transforming Growth Factor (TGF)‐Alpha and Fibroblast Growth Factor (FGF) in Human Renal Cell Carcinomas of the Clear Cell and Papillary Types,” European Journal of Cancer 36, no. 7 (2000): 932–941. 10785600 10.1016/s0959-8049(00)00030-7

[36] C. M. Li , C. E. Kim , A. A. Margolin , et al., “CTNNB1 Mutations and Overexpression of Wnt/Beta‐Catenin Target Genes in WT1‐Mutant Wilms' Tumors,” American Journal of Pathology 165, no. 6 (2004): 1943–1953. 15579438 10.1016/s0002-9440(10)63246-4 PMC1618727

[37] L. Zhao , Y. Leng , Y. Hu , et al., “Understanding Decision Curve Analysis in Clinical Prediction Model Research,” Postgraduate Medical Journal 100, no. 1185 (2024): 512–515. 38453146 10.1093/postmj/qgae027

[38] M. Iglesias‐Escudero , N. Arias‐González , and E. Martínez‐Cáceres , “Regulatory Cells and the Effect of Cancer Immunotherapy,” Molecular Cancer 22, no. 1 (2023): 26. 36739406 10.1186/s12943-023-01714-0 PMC9898962

[39] P. Bhattacharyya , R. I. Christopherson , K. K. Skarratt , J. Z. Chen , T. Balle , and S. J. Fuller , “Combination of High‐Resolution Structures for the B Cell Receptor and co‐Receptors Provides an Understanding of Their Interactions With Therapeutic Antibodies,” Cancers (Basel) 15, no. 11 (2023): 2881. 37296844 10.3390/cancers15112881 PMC10251933

[40] Z. Gong , M. Wei , A. C. Vlantis , et al., “Sodium‐Iodide Symporter and Its Related Solute Carriers in Thyroid Cancer,” Journal of Endocrinology 261, no. 1 (2024): e230373. 38329368 10.1530/JOE-23-0373

[41] X. Wei , H. Ruan , Y. Zhang , et al., “Pan‐Cancer Analysis of IFN‐γ With Possible Immunotherapeutic Significance: A Verification of Single‐Cell Sequencing and Bulk Omics Research,” Frontiers in Immunology 14 (2023): 1202150. 37646041 10.3389/fimmu.2023.1202150 PMC10461559

[42] M. G. McNamara , T. Jacobs , A. Lamarca , R. A. Hubner , J. W. Valle , and E. Amir , “Impact of High Tumor Mutational Burden in Solid Tumors and Challenges for Biomarker Application,” Cancer Treatment Reviews 89 (2020): 102084. 32738738 10.1016/j.ctrv.2020.102084

[43] E. Picard , C. P. Verschoor , G. W. Ma , and G. Pawelec , “Relationships Between Immune Landscapes, Genetic Subtypes and Responses to Immunotherapy in Colorectal Cancer,” Frontiers in Immunology 11 (2020): 369. 32210966 10.3389/fimmu.2020.00369 PMC7068608

[44] J. Yang , L. Luo , C. Zhao , et al., “A Positive Feedback Loop Between Inactive VHL‐Triggered Histone Lactylation and PDGFRβ Signaling Drives Clear Cell Renal Cell Carcinoma Progression,” International Journal of Biological Sciences 18, no. 8 (2022): 3470–3483. 35637958 10.7150/ijbs.73398 PMC9134910

[45] T. A. Chan , M. Yarchoan , E. Jaffee , et al., “Development of Tumor Mutation Burden as an Immunotherapy Biomarker: Utility for the Oncology Clinic,” Annals of Oncology 30, no. 1 (2019): 44–56. 30395155 10.1093/annonc/mdy495 PMC6336005

[46] S. Rezaei , N. Nikpanjeh , A. Rezaee , et al., “PI3K/Akt Signaling in Urological Cancers: Tumorigenesis Function, Therapeutic Potential, and Therapy Response Regulation,” European Journal of Pharmacology 955 (2023): 175909. 37490949 10.1016/j.ejphar.2023.175909

[47] Y. S. Lu , K. S. Lee , T. Y. Chao , et al., “A Phase Ib Study of Alpelisib or Buparlisib Combined With Tamoxifen Plus Goserelin in Premenopausal Women With HR‐Positive HER2‐Negative Advanced Breast Cancer,” Clinical Cancer Research 27, no. 2 (2021): 408–417. 32718997 10.1158/1078-0432.CCR-20-1008

[48] F. Rascio , F. Spadaccino , M. T. Rocchetti , et al., “The Pathogenic Role of PI3K/AKT Pathway in Cancer Onset and Drug Resistance: An Updated Review,” Cancers (Basel) 13, no. 16 (2021): 3949. 34439105 10.3390/cancers13163949 PMC8394096

[49] S. Ribback , A. Cigliano , N. Kroeger , et al., “PI3K/AKT/mTOR Pathway Plays a Major Pathogenetic Role in Glycogen Accumulation and Tumor Development in Renal Distal Tubules of Rats and Men,” Oncotarget 6, no. 15 (2015): 13036–13048. 25948777 10.18632/oncotarget.3675 PMC4536997

[50] X. Ye , J. W. Ruan , H. Huang , W. P. Huang , Y. Zhang , and F. Zhang , “PI3K‐Akt‐mTOR Inhibition by GNE‐477 Inhibits Renal Cell Carcinoma Cell Growth In Vitro and In Vivo,” Aging (Albany NY) 12, no. 10 (2020): 9489–9499. 32421688 10.18632/aging.103221 PMC7288912

[51] S. Nandi , R. Dey , A. Samadder , A. Saxena , and A. K. Saxena , “Natural Sourced Inhibitors of EGFR, PDGFR, FGFR and VEGFRMediated Signaling Pathways as Potential Anticancer Agents,” Current Medicinal Chemistry 29, no. 2 (2022): 212–234. 33655823 10.2174/0929867328666210303101345

[52] X. Qin , Z. Liu , K. Yan , Z. Fang , and Y. Fan , “Integral Analysis of the RNA Binding Protein‐Associated Prognostic Model for Renal Cell Carcinoma,” International Journal of Medical Sciences 18, no. 4 (2021): 953–963. 33456353 10.7150/ijms.50704 PMC7807188

[53] Y. Wu , X. Wei , H. Feng , et al., “Transcriptome Analyses Identify an RNA Binding Protein Related Prognostic Model for Clear Cell Renal Cell Carcinoma,” Frontiers in Genetics 11 (2021): 617872. 33488680 10.3389/fgene.2020.617872 PMC7817999

[54] K. Bi , M. X. He , Z. Bakouny , et al., “Tumor and Immune Reprogramming During Immunotherapy in Advanced Renal Cell Carcinoma,” Cancer Cell 39, no. 5 (2021): 649–661.e5. 33711272 10.1016/j.ccell.2021.02.015 PMC8115394

[55] S. Bagchi , R. Yuan , and E. G. Engleman , “Immune Checkpoint Inhibitors for the Treatment of Cancer: Clinical Impact and Mechanisms of Response and Resistance,” Annual Review of Pathology 16 (2021): 223–249. 10.1146/annurev-pathol-042020-042741 33197221

[56] H. H. Dang , H. D. K. Ta , T. T. T. Nguyen , et al., “Identifying GPSM Family Members as Potential Biomarkers in Breast Cancer: A Comprehensive Bioinformatics Analysis,” Biomedicine 9, no. 9 (2021): 1144. 10.3390/biomedicines9091144 PMC8471503 34572330

[57] T. O. Tran , L. H. T. Lam , and N. Q. K. Le , “Hyper‐Methylation of ABCG1 as an Epigenetics Biomarker in Non‐Small Cell Lung Cancer,” Functional and Integrative Genomics 23, no. 3 (2023): 256. 37523012 10.1007/s10142-023-01185-y

[58] S. Gupta , I. G. Glezerman , J. S. Hirsch , et al., “Derivation and External Validation of a Simple Risk Score for Predicting Severe Acute Kidney Injury After Intravenous Cisplatin: Cohort Study,” BMJ (Clinical Research Ed.) 384 (2024): e077169. 10.1136/bmj-2023-077169 PMC10964715 38538012

