# Supplementary figures and images for: Identification and Validation of a Prognostic Model Based on Tumour Necrosis Factor‐Related mRNAs for Kidney Renal Clear Cell Carcinoma
Source: J Cell Mol Med. 2025 Jul 17;29(14):e70657. doi: 10.1111/jcmm.70657 (PMC12268967; doi:10.1111/jcmm.70657)

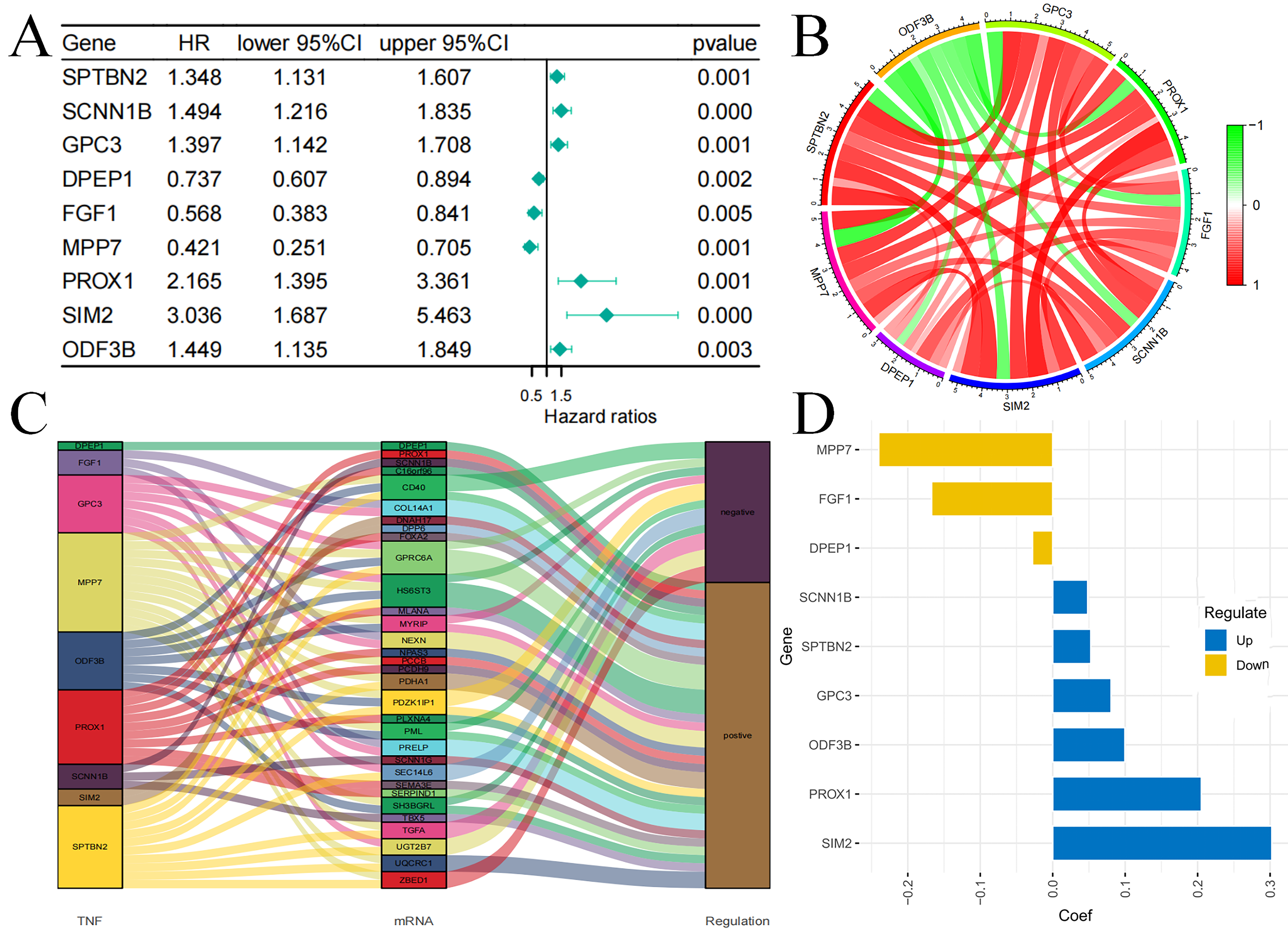

Supplement: Supplementary file 1 — Figure S1. Construct a prognostic model. Forest plots associated with multifactor regression (A); Correlation circle plot of 9 TNF‐related mRNAs (B) Mulberry plots of 9 TNF‐related mRNAs with TNF‐related genes (C) Deviation plots indicating upregulation and downregulation changes of 9 TNF‐related mRNAs (D). [file JCMM-29-e70657-s002.tif]

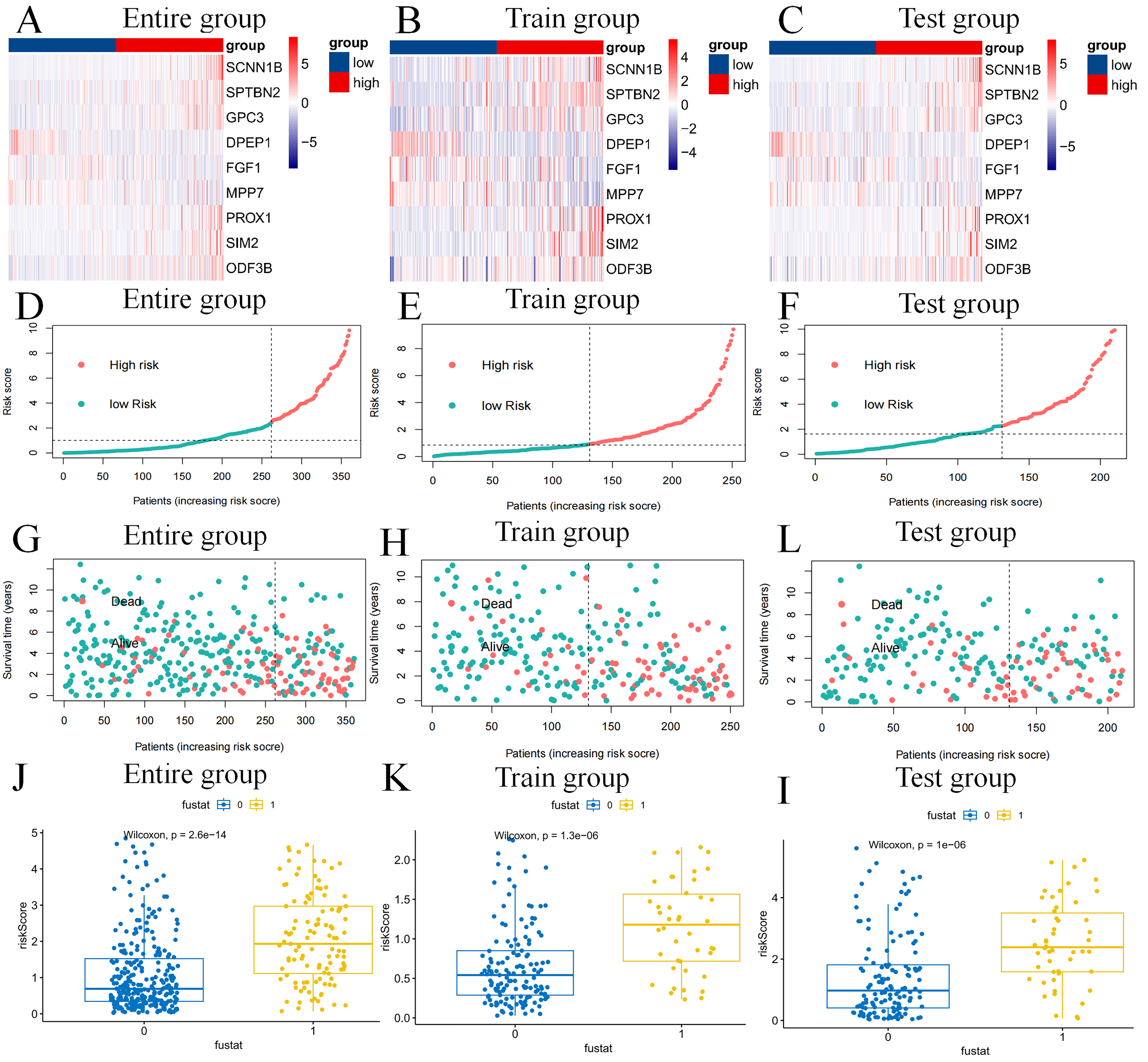

Supplement: Supplementary file 2 — Figure S2. The model prediction effect is validated by the train group, test group and the entire group. Heat map of 9 TNF‐related mRNAs expressions (A‐C). Risk curve for risk scores (D‐F) and Scatterplot (G‐I) for the survival status of each patient; Scatterplot of risk scores of patients with different survival statuses (J‐L). [file JCMM-29-e70657-s008.tif]

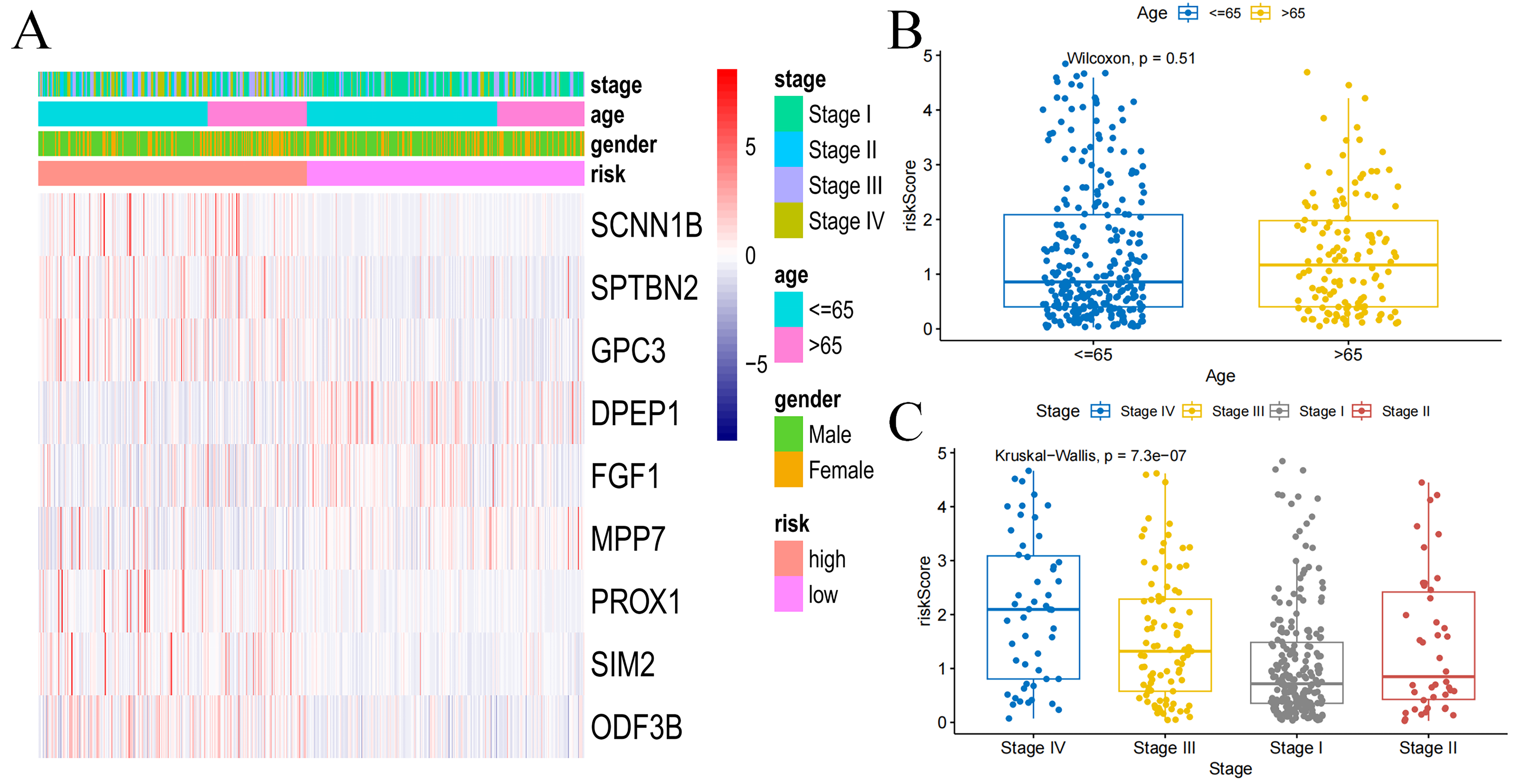

Supplement: Supplementary file 3 — Figure S3. Further validation of model effects. mRNA signature expression heat map with clinical information (A); Correlation analysis of risk signature with age (B) and stage (C). [file JCMM-29-e70657-s014.tif]

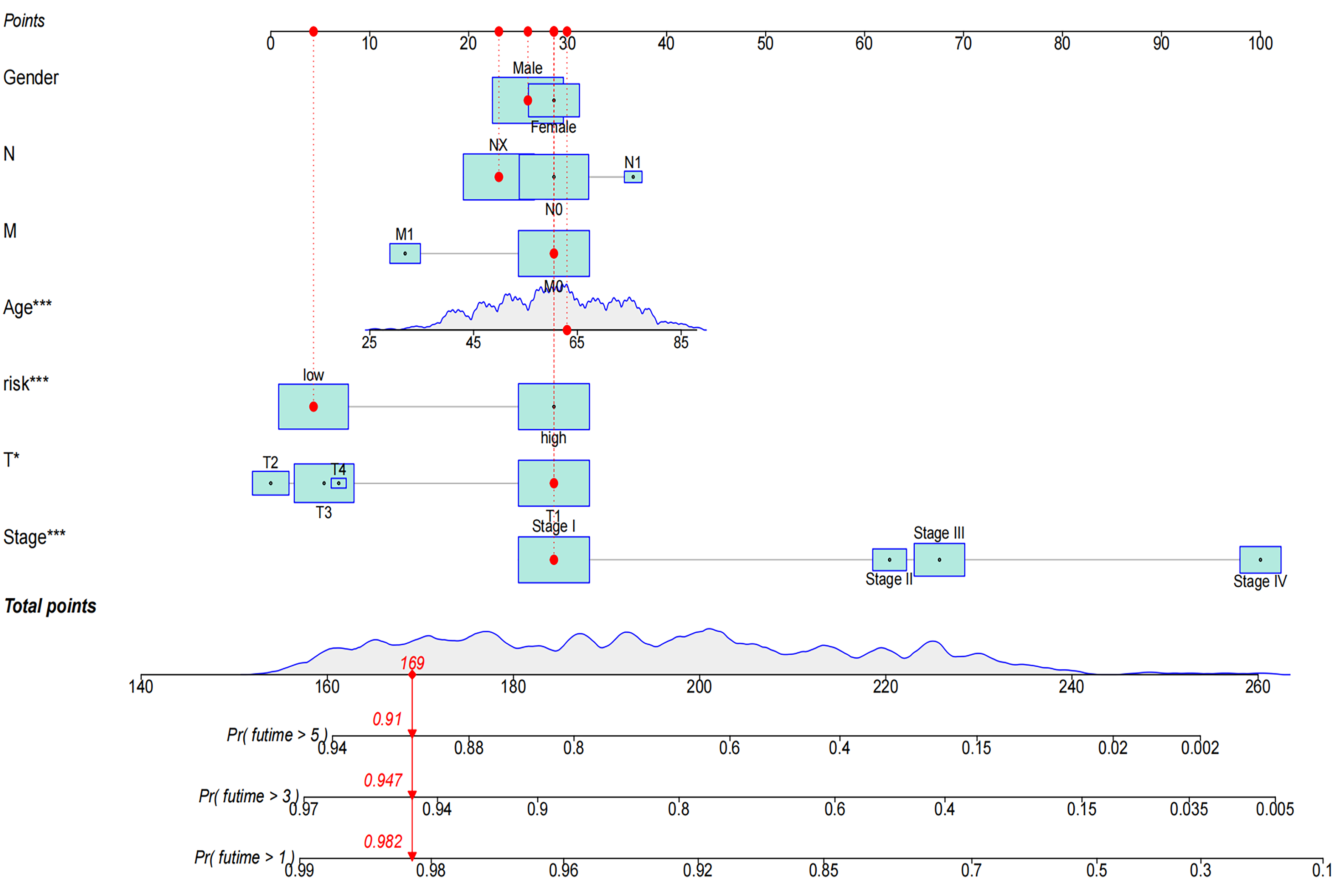

Supplement: Supplementary file 4 — Figure S4. Nomogram with patient verification. [file JCMM-29-e70657-s006.tif]

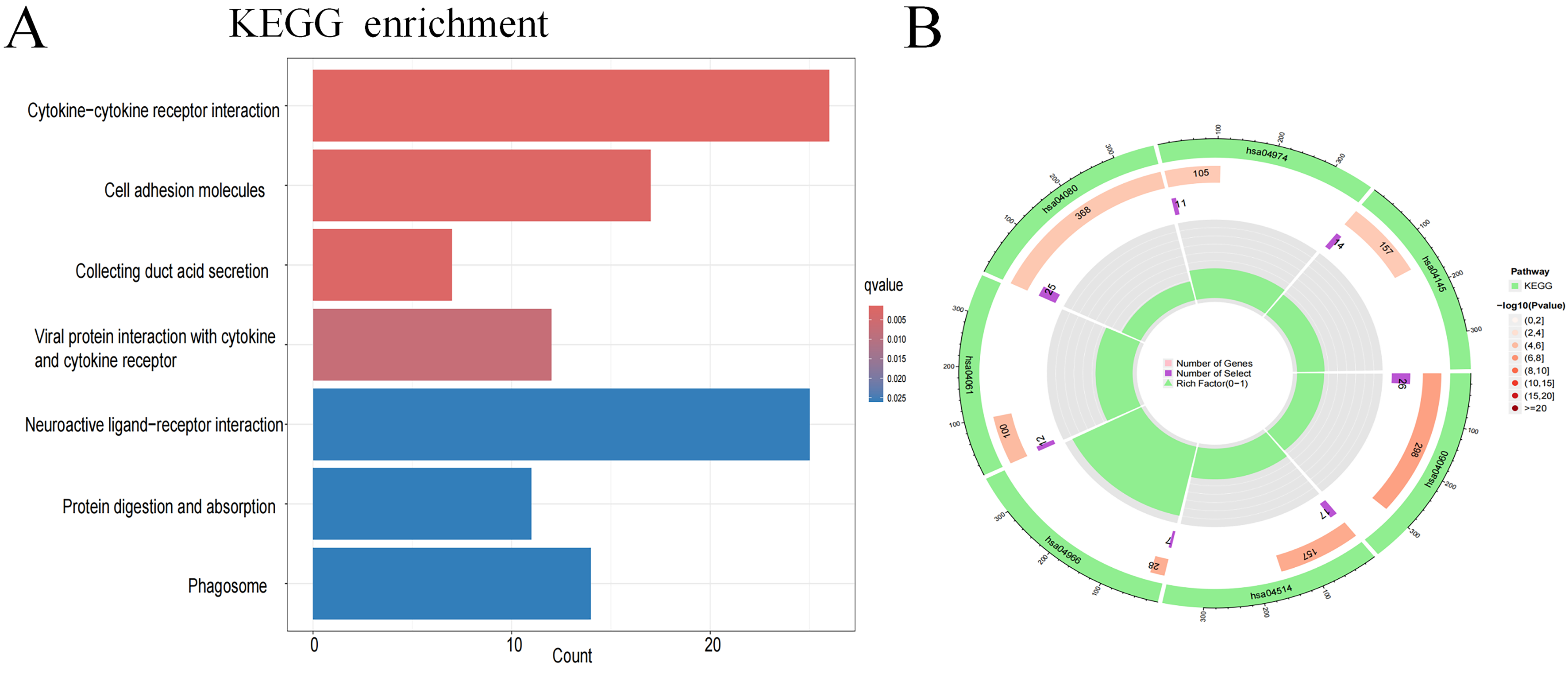

Supplement: Supplementary file 5 — Figure S5. KEGG enrichment analysis. Bar chart (A); Circle chart (B). [file JCMM-29-e70657-s015.tif]

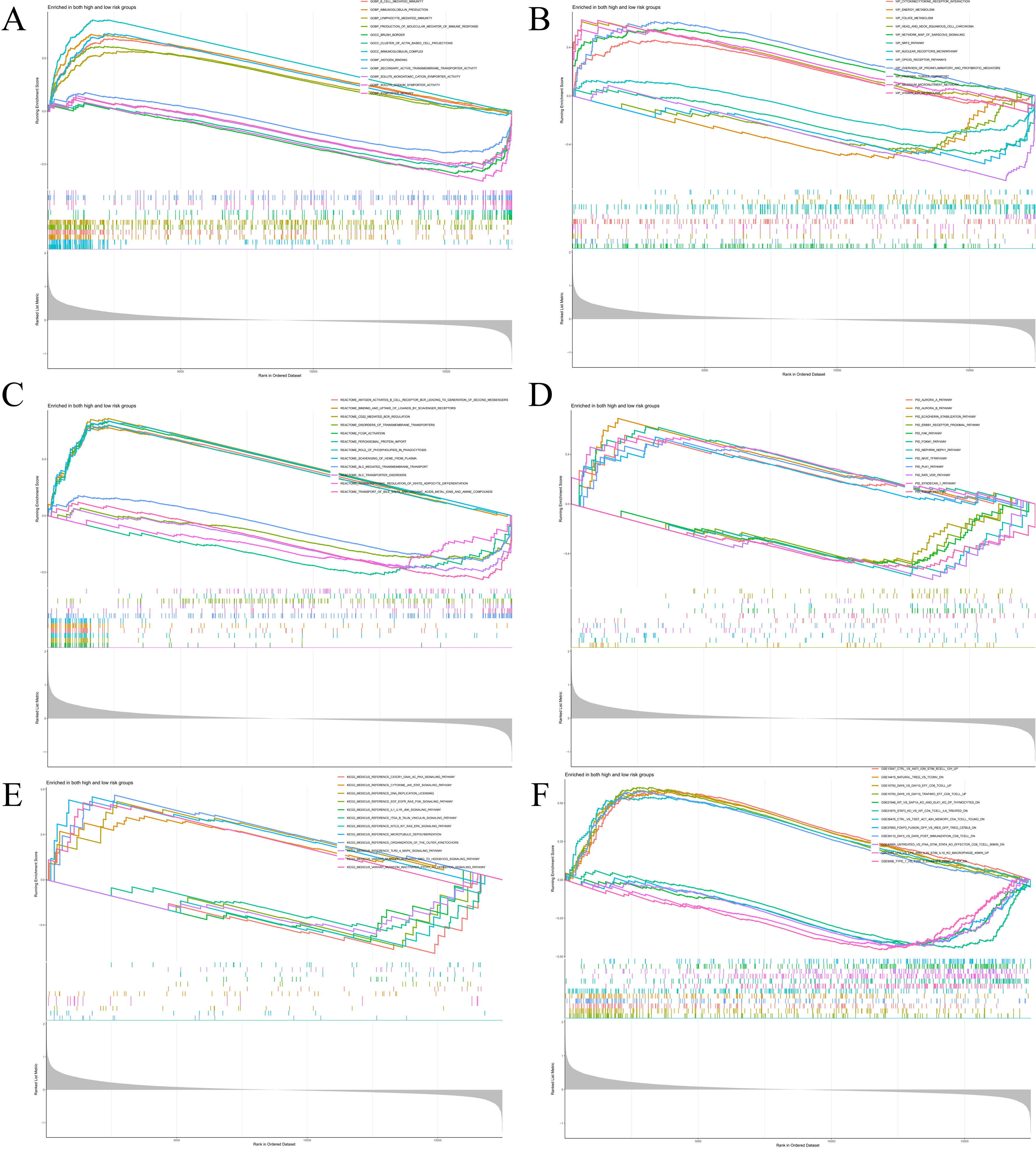

Supplement: Supplementary file 6 — Figure S6. GSEA enrichment analysis. Enrichment pathways in entire group in different gene sets (A‐F). [file JCMM-29-e70657-s012.tif]

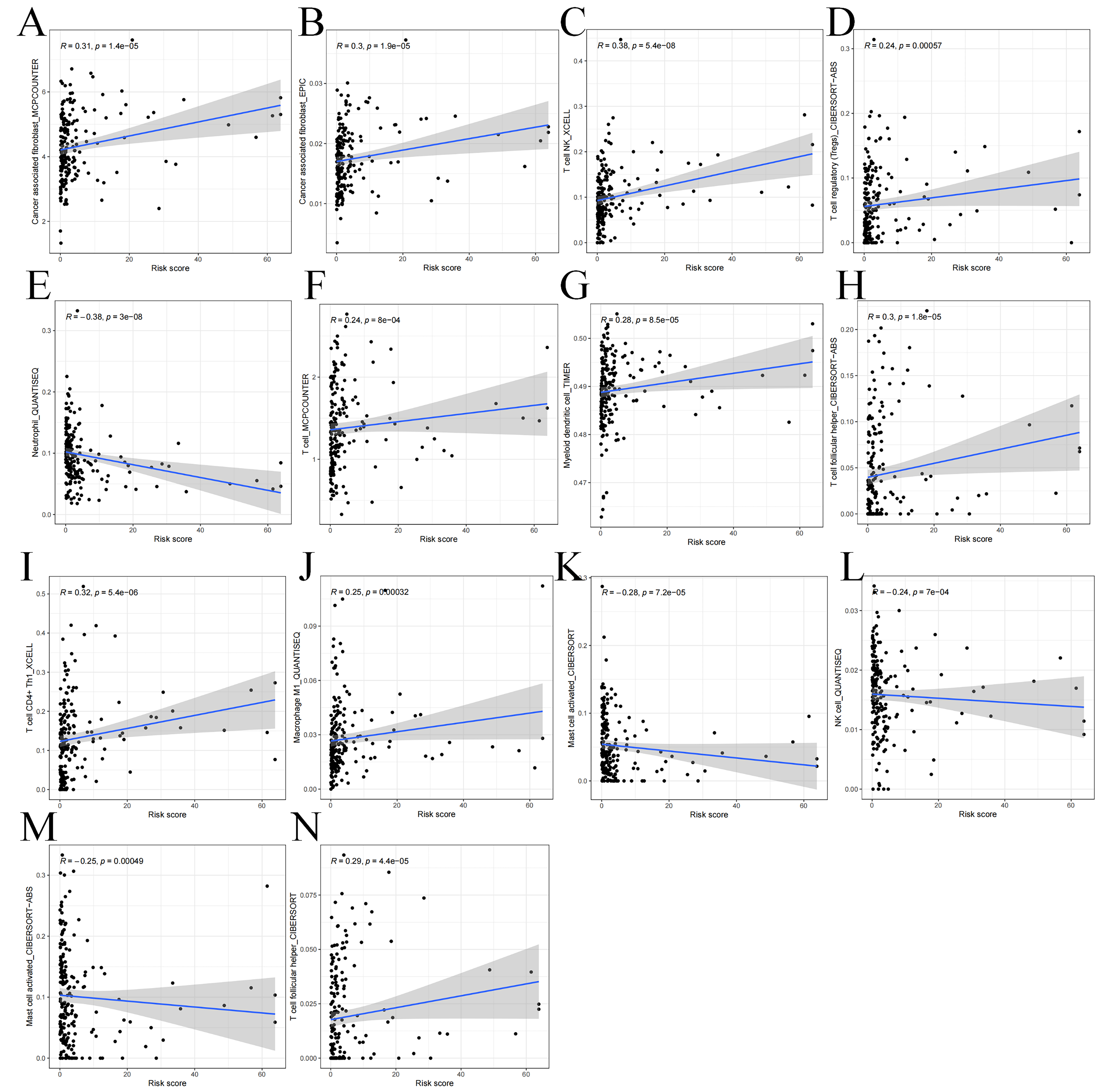

Supplement: Supplementary file 7 — Figure S7. Correlation plot of risk scores with immune cells. Scatter plot of the correlation between risk scores and cancer associated fibroblast MCPCOUNTER (A), cancer associated fibroblast EPIC (B), T cell NK (C), T cell regulatory (Tregs) (D), Neutrophil (E), T cell (F), Myeloid dendritic cell (G), T cell follicular helper (H), T cell CD4+ Th1 (I), Macrophage M1 (J), Mast cell activated (K), NK cell (L), Mast cell activated (M), T cell follicular helper (N). [file JCMM-29-e70657-s005.tif]

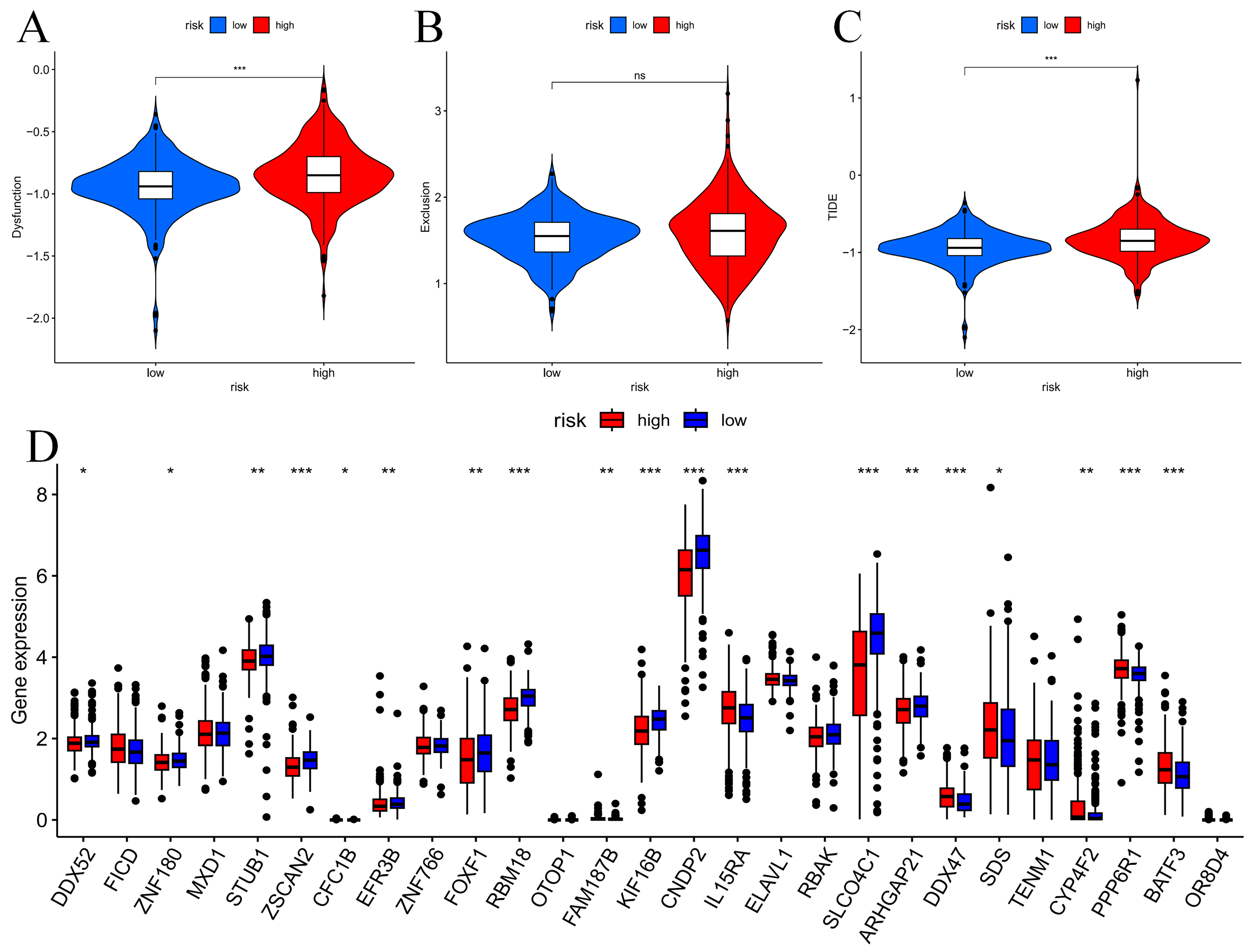

Supplement: Supplementary file 8 — Figure S8. The TNF‐related mRNAs risk pattern in tumour therapy. Differences in dysfunction (A), immune exclusion (B), TIDE (C) and immune checkpoints (D) in high and low‐risk groups. * p < 0.05, ** p < 0.01, *** p < 0.001. [file JCMM-29-e70657-s018.tif]

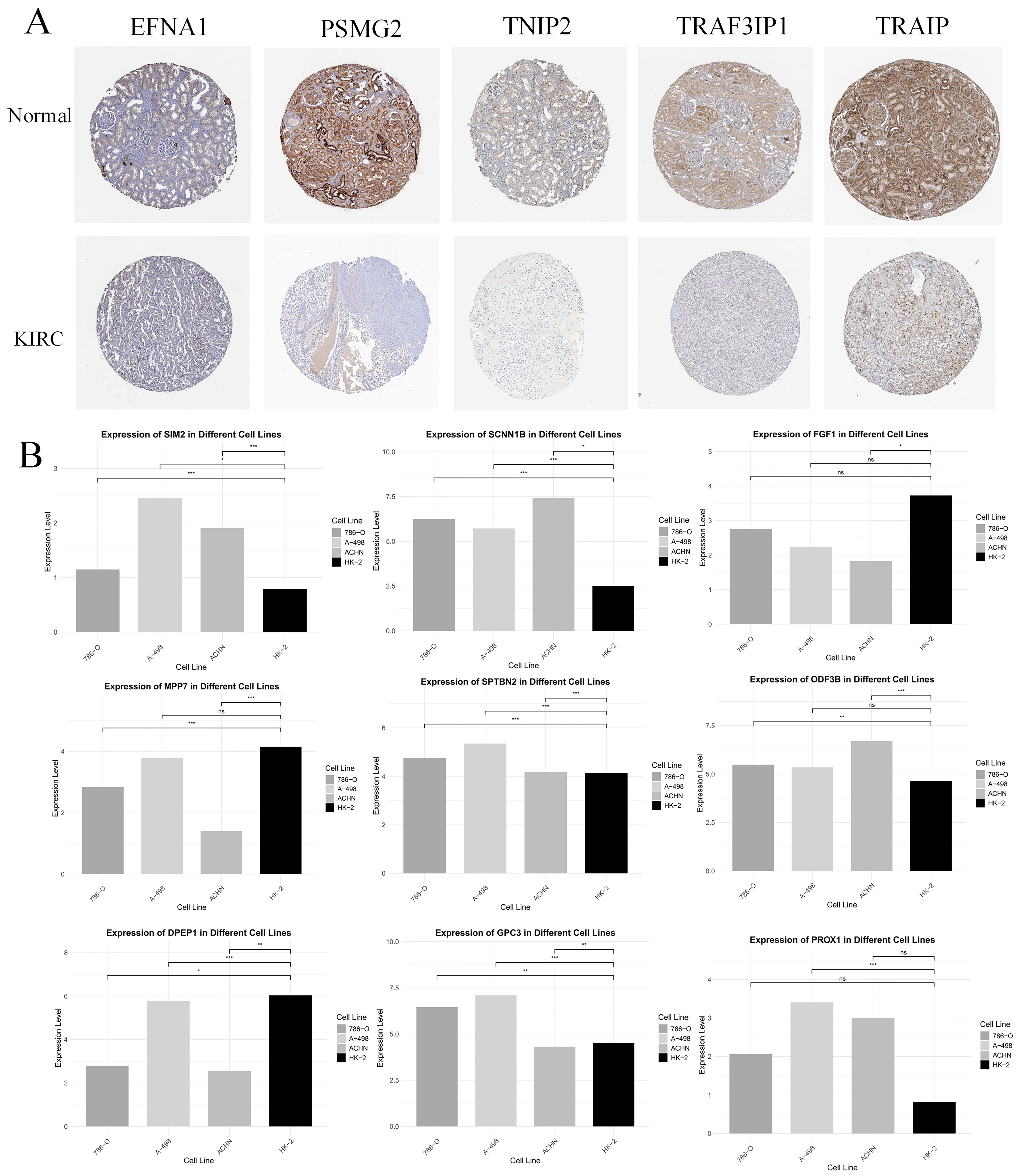

Supplement: Supplementary file 9 — Figure S9. In vitro experimental validation of the risk model. Immunohistochemical staining images of partial TNF‐related gene proteins in KIRC tissue and normal tissue (A); Relative expression of 9 TNF‐related mRNAs in different risk subgroups (B). * p < 0.05, ** p < 0.01, *** p < 0.001, **** p < 0.0001. [file JCMM-29-e70657-s001.tif]

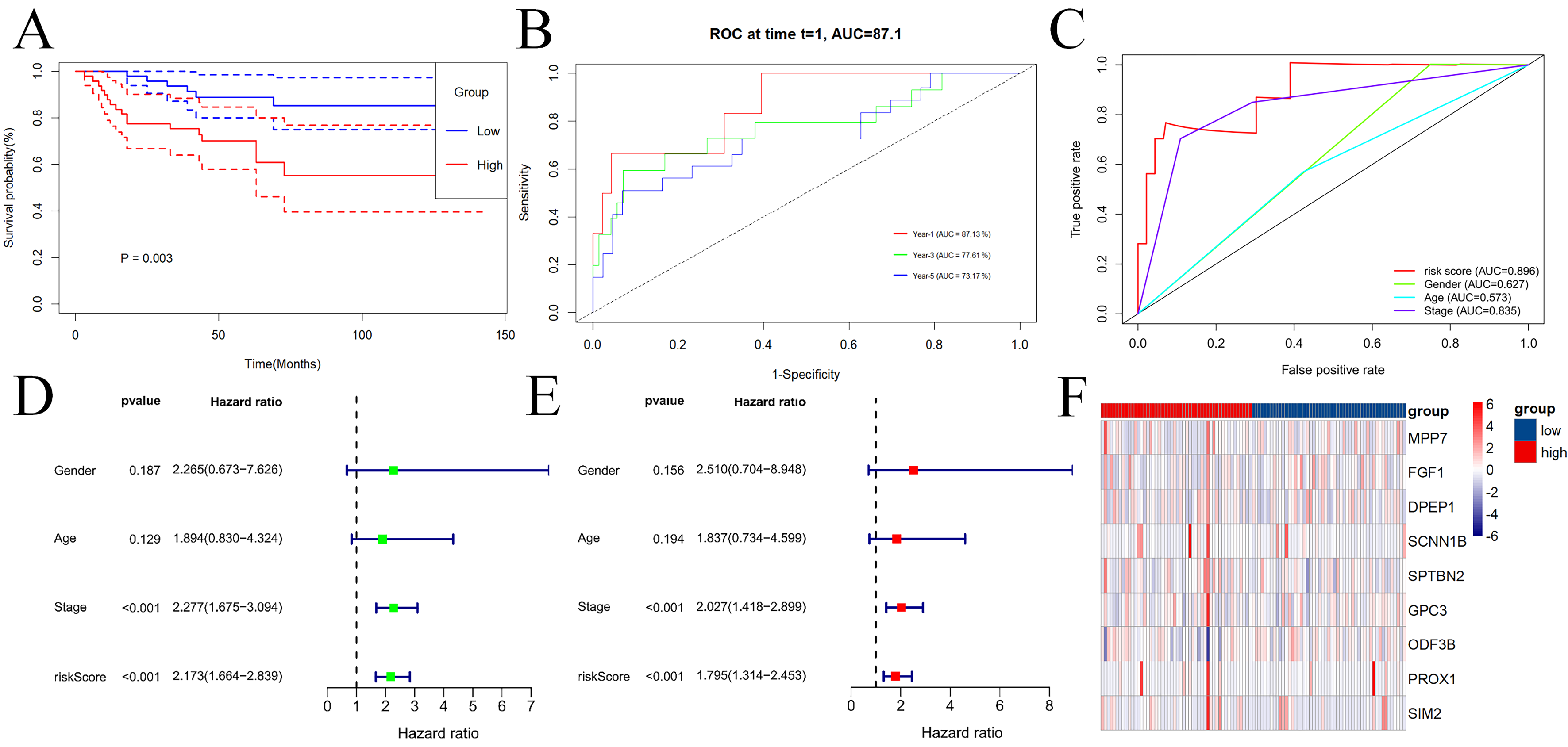

Supplement: Supplementary file 10 — Figure S10. External validation of prognostic characterisation. K‐M analysis (A) and Time‐dependent ROC curves (B) to compare the survival of the high‐risk group and low‐risk group. ROC curves containing different clinical information (C). Univariate (D) and multivariate (E) independent prognostic analysis. The heatmap between high‐risk and low‐risk group (F). [file JCMM-29-e70657-s011.tif]

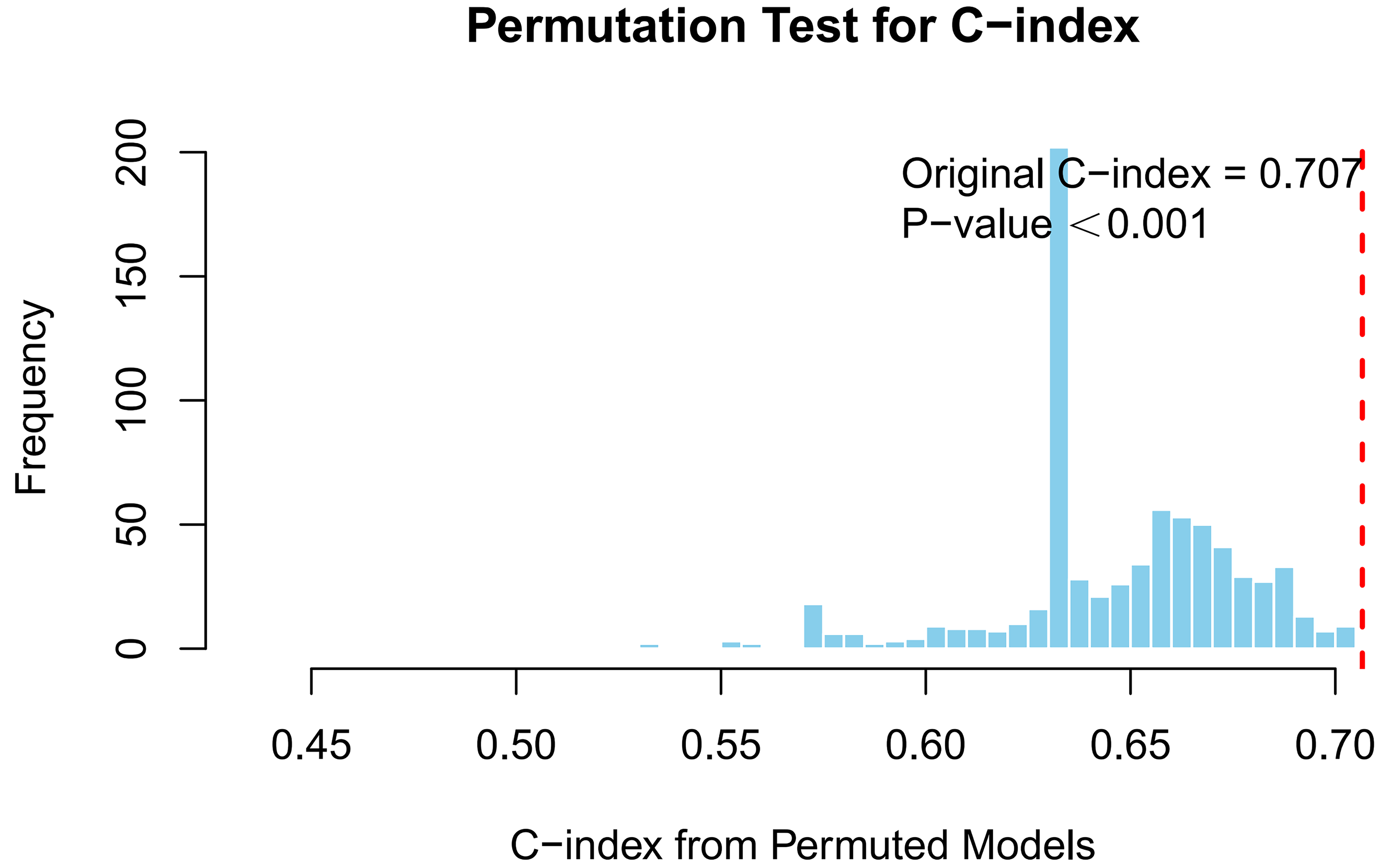

Supplement: Supplementary file 11 — Figure S11. Results of permutation testing (1000 iterations). The histogram shows the distribution of C‐index values from permuted models. The red dashed line indicates the C‐index of the original model, which lies far in the tail of the null distribution (p < 0.001), confirming that the model’s performance is unlikely to occur by chance. [file JCMM-29-e70657-s007.tif]
