# Supplementary material for: Identification and Validation of a Prognostic Model Based on Tumour Necrosis Factor‐Related mRNAs for Kidney Renal Clear Cell Carcinoma
Source: J Cell Mol Med. 2025 Jul 17;29(14):e70657. doi: 10.1111/jcmm.70657 (PMC12268967; doi:10.1111/jcmm.70657)
Supplement: Supplementary file 12 — Table S1. Primer sequences for 9 TNF‐related mRNAs. mRNA signature expression heatmap (C). [file JCMM-29-e70657-s013.docx]

**Table S1** Primer sequences for 9 TNF-related mRNAs.

| **Gene id** | **Primer F** | **Primer R** |
| --- | --- | --- |
| SCNN1B | CCCGGCTACACGTACAAGG | CCCTCACAGATGATGCGCTT |
| SPTBN2 | GAGGTCTCGCATTAAGGCTCT | CTTTGGCAGTATCTCTCCCGA |
| GPC3 | CCTTTGAAATTGTTGTTCGCCA | CCTGGGTTCATTAGCTGGGTA |
| DPEP1 | CAAGTGGCCGACCATCTGG | GGGACCCTTGGAACACCATC |
| FGF1 | CTCCCGAAGGATTAAACGACG | GTCAGTGCTGCCTGAATGCT |
| MPP7 | CCTTCCTCTGGGATATGTTTGGT | AGCCTTCACATTGGGTTTTGA |
| PROX1 | AAAGGACGGTAGGGACAGCAT | CCTTGGGGATTCATGGCACTAA |
| SIM2 | AAGGAAAATGGCGAGTTTTACGA | CGCGTCTCCTAAACCTTCGG |
| ODF3B | CCCGCCTACTCCATCTACG | TCGCTCCGGGAAGTACCTG |

**Abbreviations:** TNF: Tumor necrosis factor.
