# Supplementary material for: Identification and Validation of a Prognostic Model Based on Tumour Necrosis Factor‐Related mRNAs for Kidney Renal Clear Cell Carcinoma
Source: J Cell Mol Med. 2025 Jul 17;29(14):e70657. doi: 10.1111/jcmm.70657 (PMC12268967; doi:10.1111/jcmm.70657)
Supplement: Supplementary file 13 — Table S2. 78 differentially expressed TNF‐related mRNAs. [file JCMM-29-e70657-s016.docx]

**Table S2** 78 differentially expressed TNF-related mRNAs.

| **mRNA** | **HR (95%Cl)** | **P-value** |  | **mRNA** | **HR (95%Cl)** | **P-value** |
| --- | --- | --- | --- | --- | --- | --- |
| SCNN1G | 1.41 (1.18-1.69) | 0.00 |  | KLRG2 | 1.65 (1.11-2.45) | 0.01 |
| CLCNKA | 1.27 (1.05-1.53) | 0.01 |  | SEMA6D | 0.57 (0.37-0.87) | 0.01 |
| CASR | 0.49 (0.28-0.85) | 0.01 |  | TUBB2B | 1.51 (1.19-1.92) | 0.00 |
| SCNN1B | 1.49 (1.22-1.84) | 0.00 |  | SIM2 | 3.04 (1.69-5.46) | 0.00 |
| SPTBN2 | 1.35 (1.13-1.61) | 0.00 |  | RPS6KA6 | 0.52 (0.33-0.84) | 0.01 |
| NELL1 | 2.23 (1.29-3.87) | 0.00 |  | LRRN2 | 1.27 (1.01-1.59) | 0.04 |
| L1CAM | 1.28 (1.13-1.44) | 0.00 |  | B4GALNT3 | 1.32 (1.01-1.73) | 0.04 |
| GPC3 | 1.40 (1.14-1.71) | 0.00 |  | PLG | 0.72 (0.58-0.88) | 0.00 |
| SLC22A8 | 0.45 (0.23-0.87) | 0.02 |  | SEMA5B | 0.82 (0.70-0.96) | 0.02 |
| HS6ST2 | 1.81 (1.06-3.09) | 0.03 |  | C5orf46 | 1.17 (1.06-1.29) | 0.00 |
| HOXB9 | 1.40 (1.20-1.63) | 0.00 |  | CEL | 2.12 (1.30-3.46) | 0.00 |
| PTGER1 | 1.41 (1.00-1.98) | 0.05 |  | IYD | 0.34 (0.14-0.83) | 0.02 |
| S100A2 | 1.27 (1.06-1.53) | 0.01 |  | SCGN | 0.85 (0.77-0.93) | 0.00 |
| SOSTDC1 | 0.75 (0.58-0.98) | 0.04 |  | GABRD | 0.82 (0.66-1.00) | 0.05 |
| GATA3 | 1.27 (1.03-1.56) | 0.02 |  | ODF3B | 1.45 (1.14-1.85) | 0.00 |
| PPP1R1A | 1.21 (1.10-1.33) | 0.00 |  | NETO2 | 0.79 (0.63-0.99) | 0.04 |
| DPEP1 | 0.74 (0.61-0.89) | 0.00 |  | BRINP3 | 2.16 (1.21-3.86) | 0.01 |
| FGF1 | 0.57 (0.38-0.84) | 0.00 |  | ST8SIA4 | 0.72 (0.58-0.89) | 0.00 |
| F11 | 0.55 (0.30-0.98) | 0.04 |  | COL5A3 | 1.31 (1.04-1.66) | 0.02 |
| SUSD4 | 1.30 (1.01-1.68) | 0.04 |  | CDCA2 | 0.77 (0.62-0.94) | 0.01 |
| MT1G | 1.11 (1.01-1.23) | 0.04 |  | GZMH | 1.25 (1.02-1.54) | 0.04 |
| RALYL | 2.36 (1.04-5.35) | 0.04 |  | SLC17A4 | 0.83 (0.74-0.94) | 0.00 |
| CRHBP | 0.64 (0.43-0.95) | 0.03 |  | PCSK6 | 0.82 (0.69-0.98) | 0.03 |
| NDNF | 1.14 (1.01-1.29) | 0.04 |  | DIRAS2 | 0.69 (0.57-0.82) | 0.00 |
| SLC4A11 | 1.61 (1.07-2.41) | 0.02 |  | TMEM45A | 1.30 (1.12-1.51) | 0.00 |
| DNER | 1.19 (1.00-1.42) | 0.05 |  | ITGAX | 1.32 (1.02-1.70) | 0.03 |
| FAM167A | 1.49 (1.10-2.02) | 0.01 |  | QRFPR | 0.65 (0.54-0.77) | 0.00 |
| TMEM178A | 1.44 (1.10-1.90) | 0.01 |  | LAIR1 | 1.30 (1.00-1.69) | 0.05 |
| AFM | 0.47 (0.26-0.86) | 0.01 |  | HRH2 | 0.76 (0.65-0.89) | 0.00 |
| ESRRG | 0.49 (0.32-0.73) | 0.00 |  | PTHLH | 1.19 (1.08-1.31) | 0.00 |
| COL23A1 | 0.85 (0.75-0.95) | 0.01 |  | SCGB3A2 | 1.29 (1.00-1.66) | 0.05 |
| SLC52A3 | 1.49 (1.14-1.95) | 0.00 |  | RASD2 | 1.39 (1.05-1.85) | 0.02 |
| CLIC5 | 0.59 (0.43-0.81) | 0.00 |  | GDF6 | 0.73 (0.60-0.88) | 0.00 |
| ENPP3 | 0.87 (0.79-0.97) | 0.01 |  | CXCL13 | 1.27 (1.12-1.43) | 0.00 |
| ATP6V1C2 | 1.33 (1.10-1.62) | 0.00 |  | PMCH | 1.80 (1.27-2.56) | 0.00 |
| SLC6A3 | 0.86 (0.79-0.95) | 0.00 |  | PAEP | 1.24 (1.12-1.38) | 0.00 |
| TNFAIP6 | 0.89 (0.79-1.00) | 0.05 |  | SLC18A3 | 1.26 (1.12-1.42) | 0.00 |
| MPP7 | 0.42 (0.25-0.71) | 0.00 |  | PROX1 | 2.17 (1.40-3.36) | 0.00 |
| CYP2J2 | 0.85 (0.77-0.94) | 0.00 |  | SYT7 | 1.37 (1.04-1.81) | 0.02 |

**Abbreviations:** TNF: Tumor necrosis factor; HR: Hazard Ratio; CL: Confidence level.
