# Supplementary material for: Identification and Validation of a Prognostic Model Based on Tumour Necrosis Factor‐Related mRNAs for Kidney Renal Clear Cell Carcinoma
Source: J Cell Mol Med. 2025 Jul 17;29(14):e70657. doi: 10.1111/jcmm.70657 (PMC12268967; doi:10.1111/jcmm.70657)
Supplement: Supplementary file 14 — Table S3. 20 differentially expressed TNF‐related mRNAs. [file JCMM-29-e70657-s017.docx]

**Table S3** 20 differentially expressed TNF-related mRNAs.

| **mRNA** | **Coef** |
| --- | --- |
| SCNN1G | 0.0975 |
| CASR | -0.0736 |
| SCNN1B | 0.0483 |
| SPTBN2 | 0.0525 |
| GPC3 | 0.0805 |
| HOXB9 | 0.1090 |
| DPEP1 | -0.0279 |
| FGF1 | -0.1671 |
| ESRRG | -0.0414 |
| CLIC5 | -0.0319 |
| MPP7 | -0.2400 |
| PROX1 | 0.2051 |
| SIM2 | 0.3024 |
| RPS6KA6 | -0.0156 |
| ODF3B | 0.0996 |
| ST8SIA4 | -0.0192 |
| QRFPR | -0.1775 |
| PTHLH | 0.0025 |
| CXCL13 | 0.0299 |
| PAEP | 0.0236 |

**Abbreviations:** TNF: Tumor necrosis factor.
