# Supplementary material for: Identification and Validation of a Prognostic Model Based on Tumour Necrosis Factor‐Related mRNAs for Kidney Renal Clear Cell Carcinoma
Source: J Cell Mol Med. 2025 Jul 17;29(14):e70657. doi: 10.1111/jcmm.70657 (PMC12268967; doi:10.1111/jcmm.70657)
Supplement: Supplementary file 15 — Table S4. GSEA pathways for different risk groups. [file JCMM-29-e70657-s009.docx]

**Table S4** GSEA pathways for different risk groups.

| **Pathways** | **Group** |
| --- | --- |
| **c2.cp.wikipathways.v2024.1.Hs.symbols.gmt** |  |
| WP_PROXIMAL_TUBULE_TRANSPORT | Low risk |
| WP_OPIOID_RECEPTOR_PATHWAYS | Low risk |
| WP_NRF2_PATHWAY | Low risk |
| WP_NUCLEAR_RECEPTORS_METAPATHWAY | Low risk |
| WP_HEAD_AND_NECK_SQUAMOUS_CELL_CARCINOMA | Low risk |
| WP_ENERGY_METABOLISM | Low risk |
| WP_OVERVIEW_OF_PROINFLAMMATORY_AND_PROFIBROTIC_MEDIATORS | High risk |
| WP_NETWORK_MAP_OF_SARSCOV2_SIGNALING | High risk |
| WP_SELENIUM_MICRONUTRIENT_NETWORK | High risk |
| WP_FOLATE_METABOLISM | High risk |
| WP_VITAMIN_B12_METABOLISM | High risk |
| WP_CYTOKINECYTOKINE_RECEPTOR_INTERACTION | High risk |
| **c5.go.v2024.1.Hs.symbols.gmt** |  |
| GOMF_SOLUTE_SODIUM_SYMPORTER_ACTIVITY | Low risk |
| NSE GOCC_BRUSH_BORDER | Low risk |
| ITY GOMF_SOLUTE_MONOATOMIC_CATION_SYMPORTER_ACTIVITY | Low risk |
| GOCC_CLUSTER_OF_ACTIN_BASED_CELL_PROJECTIONS | Low risk |
| GOMF_SYMPORTER_ACTIVITY | Low risk |
| GOMF_SECONDARY_ACTIVE_TRANSMEMBRANE_TRANSPORTER_ACTIVITY | Low risk |
| GOCC_IMMUNOGLOBULIN_COMPLEX | High risk |
| GOBP_IMMUNOGLOBULIN_PRODUCTION | High risk |
| GOMF_ANTIGEN_BINDING | High risk |
| GOBP_B_CELL_MEDIATED_IMMUNITY | High risk |
| GOBP_PRODUCTION_OF_MOLECULAR_MEDIATOR_OF_IMMUNE_RESPONSE | High risk |
| GOBP_LYMPHOCYTE_MEDIATED_IMMUNITY | High risk |
| **c7.immunesigdb.v2024.1.Hs.symbols.gmt** |  |
| GSE9006_TYPE_1_VS_TYPE_2_DIABETES_PBMC_AT_DX_DN | Low risk |
| GSE5589_LPS_VS_LPS_AND_IL10_STIM_IL10_KO_MACROPHAGE_45MIN_UP | Low risk |
| GSE40666_UNTREATED_VS_IFNA_STIM_STAT4_KO_EFFECTOR_CD8_TCELL_90MIN_DN | Low risk |
| GSE21546_WT_VS_SAP1A_KO_AND_ELK1_KO_DP_THYMOCYTES_DN | Low risk |
| GSE37605_FOXP3_FUSION_GFP_VS_IRES_GFP_TREG_C57BL6_DN | Low risk |
| GSE21670_STAT3_KO_VS_WT_CD4_TCELL_IL6_TREATED_DN | Low risk |
| GSE13547_CTRL_VS_ANTI_IGM_STIM_BCELL_12H_UP | High risk |
| GSE14415_NATURAL_TREG_VS_TCONV_DN | High risk |
| GSE15750_DAY6_VS_DAY10_EFF_CD8_TCELL_UP | High risk |
| GSE15750_DAY6_VS_DAY10_TRAF6KO_EFF_CD8_TCELL_UP | High risk |
| GSE36476_CTRL_VS_TSST_ACT_40H_MEMORY_CD4_TCELL_YOUNG | High risk |
| GSE39110_DAY3_VS_DAY6_POST_IMMUNIZATION_CD8_TCELL_DN | High risk |
| **c2.cp.kegg_medicus.v2024.1.Hs.symbols.gmt** |  |
| KEGG_MEDICUS_REFERENCE_CX3CR1_GNAI_AC_PKA_SIGNALING_PATHWAY | Low risk |
| KEGG_MEDICUS_REFERENCE_KITLG_KIT_RAS_ERK_SIGNALING_PATHWAY | Low risk |
| KEGG_MEDICUS_REFERENCE_EGF_EGFR_RAS_PI3K_SIGNALING_PATHWAY | Low risk |
| KEGG_MEDICUS_REFERENCE_ITGA_B_TALIN_VINCULIN_SIGNALING_PATHWAY | Low risk |
| KEGG_MEDICUS_REFERENCE_TLR2_4_MAPK_SIGNALING_PATHWAY | Low risk |
| KEGG_MEDICUS_REFERENCE_IL1_IL1R_JNK_SIGNALING_PATHWAY | Low risk |
| KEGG_MEDICUS_REFERENCE_CYTOKINE_JAK_STAT_SIGNALING_PATHWAY | High risk |
| KEGG_MEDICUS_REFERENCE_DNA_REPLICATION_LICENSING | High risk |
| KEGG_MEDICUS_REFERENCE_MICROTUBULE_DEPOLYMERIZATION | High risk |
| KEGG_MEDICUS_REFERENCE_ORGANIZATION_OF_THE_OUTER_KINETOCHORE | High risk |
| KEGG_MEDICUS_VARIANT_MUTATION_ACTIVATED_SMO_TO_HEDGEHOG_SIGNALING_PATHWAY | High risk |
| KEGG_MEDICUS_VARIANT_MUTATION_INACTIVATED_PTCH1_TO_HEDGEHOG_SIGNALING_PATHWAY | High risk |
| **c2.cp.pid.v2024.1.Hs.symbols.gmt** |  |
| PID_RXR_VDR_PATHWAY | Low risk |
| PID_NEPHRIN_NEPH1_PATHWAY | Low risk |
| PID_TGFBR_PATHWAY | Low risk |
| PID_FAK_PATHWAY | Low risk |
| PID_ERBB1_RECEPTOR_PROXIMAL_PATHWAY | Low risk |
| PID_ECADHERIN_STABILIZATION_PATHWAY | Low risk |
| PID_AURORA_A_PATHWAY | High risk |
| PID_AURORA_B_PATHWAY | High risk |
| PID_NFAT_TFPATHWAY | High risk |
| PID_FOXM1_PATHWAY | High risk |
| PID_PLK1_PATHWAY | High risk |
| PID_SYNDECAN_1_PATHWAY | High risk |
| **c2.cp.reactome.v7.0.symbols.gmt** |  |
| REACTOME_TRANSPORT_OF_BILE_SALTS_AND_ORGANIC_ACIDS_METAL_IONS_AND_AMINE_COMPOUNDS | Low risk |
| REACTOME_SLC_TRANSPORTER_DISORDERS | Low risk |
| REACTOME_SLC_MEDIATED_TRANSMEMBRANE_TRANSPORT | Low risk |
| REACTOME_DISORDERS_OF_TRANSMEMBRANE_TRANSPORTERS | Low risk |
| REACTOME_PEROXISOMAL_PROTEIN_IMPORT | Low risk |
| REACTOME_TRANSCRIPTIONAL_REGULATION_OF_WHITE_ADIPOCYTE_DIFFERENTIATION | Low risk |
| REACTOME_ANTIGEN_ACTIVATES_B_CELL_RECEPTOR_BCR_LEADING_TO_GENERATION_OF_SECOND_MESSENGERS | High risk |
| REACTOME_BINDING_AND_UPTAKE_OF_LIGANDS_BY_SCAVENGER_RECEPTORS | High risk |
| REACTOME_CD22_MEDIATED_BCR_REGULATION | High risk |
| REACTOME_FCGR_ACTIVATION | High risk |
| REACTOME_ROLE_OF_PHOSPHOLIPIDS_IN_PHAGOCYTOSIS | High risk |
| REACTOME_SCAVENGING_OF_HEME_FROM_PLASMA | High risk |

**Abbreviations:** GSEA: Gene Set Enrichment Analysis.
