# Supplementary material for: Identification and Validation of a Prognostic Model Based on Tumour Necrosis Factor‐Related mRNAs for Kidney Renal Clear Cell Carcinoma
Source: J Cell Mol Med. 2025 Jul 17;29(14):e70657. doi: 10.1111/jcmm.70657 (PMC12268967; doi:10.1111/jcmm.70657)
Supplement: Supplementary file 16 — Table S5. Antineoplastic drug sensitivity information (sensitive group: low). [file JCMM-29-e70657-s003.docx]

**Table S5** Antineoplastic drug sensitivity information (sensitive group: low).

| **Target pathways** | **Low-risk group** |  | **High-risk group** | **P-value** |
| --- | --- | --- | --- | --- |
|  | **IC50 (25%-75%）** |  | **IC50 (25%-75%）** |  |
| **WNT signaling** |  |  |  |  |
| SB216763 | 188.20(166.88-210.60) |  | 192.76(166.06-227.41) | 0.04 |
| **RTK signaling** |  |  |  |  |
| Carmustine | 420.34(349.77-500.54) |  | 455.65(358.66-585.30) | 0.00 |
| **Protein stability and degradation** | |  |  |  |
| NVP.ADW742 13.19(10.87-18.69) | |  | 15.63(12.04-21.50) | 0.00 |
| **Other, kinases** |  |  |  |  |
| Sinularin | 32.22(27.66-40.61) |  | 36.22(28.80-45.70) | 0.00 |
| **Other** |  |  |  |  |
| LY2109761 | 155.71(126.24-198.36) |  | 173.73(138.44-244.59) | 0.00 |
| Ibrutinib | 78.50(62.92-104.20) |  | 92.92(72.19-133.53) | 0.00 |
| **Mitosis** |  |  |  |  |
| OF.1 | 58.46(50.55-68.32) |  | 63.92(49.86-81.39) | 0.00 |
| **Genome integrity** |  |  |  |  |
| NU7441 | 12.95(11.80-14.35) |  | 13.70(12.14-15.81) | 0.00 |
| **ERK MAPK signaling** |  |  |  |  |
| Cediranib | 7.64(6.68-9.30) |  | 8.29(6.92-10.90) | 0.00 |
| **EGFR signaling** |  |  |  |  |
| AZD3759 | 13.47(11.23-16.12) |  | 15.70(12.57-19.04) | 0.00 |
| **Cell cycle** |  |  |  |  |
| Osimertinib | 5.07(3.86-6.28) |  | 6.04(4.56-8.27) | 0.00 |
| **JNK and p38 signaling** |  |  |  |  |
| Doramapimod | 88.53(79.17-97.15) |  | 91.71(81.67-105.24) | 0.01 |

**Abbreviation:** IC50: half maximal inhibitory concentration.
