# Supplementary material for: Identification and Validation of a Prognostic Model Based on Tumour Necrosis Factor‐Related mRNAs for Kidney Renal Clear Cell Carcinoma
Source: J Cell Mol Med. 2025 Jul 17;29(14):e70657. doi: 10.1111/jcmm.70657 (PMC12268967; doi:10.1111/jcmm.70657)
Supplement: Supplementary file 17 — Table S6. Antineoplastic drug sensitivity information (sensitive group: high). [file JCMM-29-e70657-s004.docx]

**Table S6** Antineoplastic drug sensitivity information (sensitive group: high).

| **Target pathway** | **Low-risk group** |  | **High-risk group** | **P-value** |
| --- | --- | --- | --- | --- |
|  | **IC50 (25%-75%）** |  | **IC50 (25%-75%）** |  |
| **WNT signaling** |  |  |  |  |
| WIKI4 | 42.16(37.36-48.07) |  | 38.87(33.64-44.62) | 0.00 |
| VX.11e | 17.43(13.70-21.91) |  | 16.17(11.97-21.84) | 0.03 |
| LGK974 | 58.00(47.85-67.80) |  | 52.85(42.46-67.56) | 0.01 |
| **Unclassified** |  |  |  |  |
| Sepantronium.bromide | 0.01(0.01-0.02) |  | 0.01(0.01-0.02) | 0.04 |
| Elephantin | 34.56(28.31-42.50) |  | 27.77(22.03-38.49) | 0.00 |
| **RTK signaling** |  |  |  |  |
| Oxaliplatin_1089 | 44.66(32.86-59.45) |  | 39.36(24.96-61.21) | 0.01 |
| Sabutoclax | 0.70(0.57-0.88) |  | 0.64(0.50-0.80) | 0.00 |
| Oxaliplatin_1806 | 158.56(119.38-200.53) |  | 138.87(99.60-200.21) | 0.03 |
| Axitinib | 21.59(18.83-25.09) |  | 20.18(17.21-24.93) | 0.01 |
| AZD1332 | 48.78(38.89-59.30) |  | 41.10(38.89-59.30) | 0.00 |
| **PI3K/MTOR signaling** | |  |  |  |
| Uprosertib_2106 17.31(12.48-23.06) | |  | 14.67(10.58-22.90) | 0.02 |
| Podophyllotoxin.bromide 0.51(0.42-0.69) | |  | 0.47(0.35-0.65) | 0.00 |
| BDP.00009066 10.56(9.04-12.51) | |  | 9.82(7.88-12.02) | 0.00 |
| AGI.5198 107.20(97.44-120.17) | |  | 98.93(85.14-117.40) | 0.00 |
| Afuresertib 12.66(10.13-16.14) | |  | 11.57(9.07-15.93) | 0.04 |
| Dactolisib 0.21(0.16-0.26) | |  | 0.18(0.13-0.24) | 0.00 |
| Pictilisib 4.31(3.43-5.22) | |  | 3.54(2.81-4.75) | 0.00 |
| Uprosertib_2106 17.31(12.48-23.06) | |  | 14.67(10.58-22.90) | 0.02 |
| Buparlisib 2.56(2.20-2.98) | |  | 2.44(2.03-2.94) | 0.01 |
| Ipatasertib 34.79(27.57-45.53) | |  | 31.04(24.36-41.57) | 0.01 |
| **Other, kinases** |  |  |  |  |
| JAK_8517 | 19.87(15.11-28.40) |  | 16.85(12.83-28.47) | 0.04 |
| Ipatasertib_1924 | 34.79(27.57-45.53) |  | 31.04(24.36-41.57) | 0.01 |
| AZD5153 | 5.46(4.30-7.15) |  | 4.94(3.63-6.93) | 0.01 |
| JAK_8517 | 19.87(15.11-28.40) |  | 16.85(12.83-28.47) | 0.04 |
| **Other** |  |  |  |  |
| VSP34_8731 | 10.62(9.00-14.01) |  | 9.89(7.92-12.71) | 0.00 |
| Dactinomycin_1911 | 0.01(0.01-0.01) |  | 0.01(0.00-0.01) | 0.00 |
| Eg5_9814 | 0.04(0.03-0.06) |  | 0.04(0.03-0.06) | 0.03 |
| **Mitosis** |  |  |  |  |
| Eg5_9814_1712 | 0.04(0.03-0.06) |  | 0.04(0.03-0.06) | 0.03 |
| Vincristine | 0.18(0.11-0.28) |  | 0.13(0.08-0.26) | 0.00 |
| VE821 | 63.98(46.90-85.26) |  | 54.98(35.35-81.88) | 0.00 |
| Docetaxel_1007 | 0.01(0.01-0.01) |  | 0.01(0.01-0.01) | 0.00 |
| Docetaxel_1819 | 0.1(0.06-0.16) |  | 0.08(0.04-0.16) | 0.00 |
| **Hormone-related** |  |  |  |  |
| Teniposide | 1.83(1.17-2.72) |  | 1.34(0.76-2.46) | 0.00 |
| GDC0810 | 140.07(120.83-159.44) |  | 133.08(104.58-159.24) | 0.03 |
| Fulvestrant_1200 | 18.56(15.69-22.75) |  | 17.64(14.30-22.00) | 0.04 |
| **Genome integrity** |  |  |  |  |
| Telomerase.Inhibitor.IX | 1.66(1.41-2.11) |  | 1.41(1.10-1.92) | 0.00 |
| MK.8776 | 26.08(18.97-34.17) |  | 21.26(14.65-33.18) | 0.00 |
| MIM1 | 51.17(39.32-63.96) |  | 47.18(35.49-61.38) | 0.01 |
| AZD6738 | 8.75(6.42-11.99) |  | 6.17(4.26-9.61) | 0.00 |
| VE821 | 63.98(46.90-85.26) |  | 54.98(35.35-81.88) | 0.00 |
| **ERK MAPK signaling** | |  |  |  |
| ULK1_4989 11.39(7.98-16.23) | |  | 8.05(5.20-12.42) | 0.00 |
| **EGFR signaling** |  |  |  |  |
| GNE.317 | 1.79(1.54-2.10) |  | 1.54(1.27-1.88) | 0.00 |
| Ribociclib | 45.49(39.43-50.58) |  | 42.38(35.49-48.94) | 0.00 |
| **DNA replication** |  |  |  |  |
| Topotecan | 1.31(0.91-1.96) |  | 0.84(0.54-1.40) | 0.00 |
| Mitoxantrone | 1.83(1.25-2.82) |  | 1.65(0.94-2.82) | 0.02 |
| CDK9_5038 | 0.10(0.08-0.15) |  | 0.08(0.06-0.13) | 0.00 |
| MIRA.1 | 231.61(175.99-301.98) |  | 212.98(149.70-297.11) | 0.05 |
| IRAK4_4710 | 142.66(125.72-159.70) |  | 134.83(112.55-157.60) | 0.00 |
| GSK2578215A | 139.39(123.08-157.51) |  | 128.36(105.98-156.81) | 0.00 |
| BMS.754807 | 1.43(1.13-1.97) |  | 1.28(0.92-2.01) | 0.02 |
| Oxaliplatin_1089 | 44.66(32.86-59.45) |  | 39.36(24.96-61.21) | 0.01 |
| Oxaliplatin_1806 | 158.56(119.38-200.53) |  | 138.87(99.60-200.21) | 0.03 |
| Temozolomide | 406.53(310.99-529.04) |  | 369.09(268.47-515.59) | 0.03 |
| **Chromatin other** |  |  |  |  |
| ERK_6604 | 33.40(26.42-42.97) |  | 29.68(21.21-38.60) | 0.00 |
| Vinorelbine | 0.05(0.03-0.09) |  | 0.03(0.02-0.06) | 0.00 |
| Entospletinib | 40.75(33.74-49.18) |  | 37.73(30.38-48.66) | 0.10 |
| **Cell cycle** |  |  |  |  |
| PRT062607 | 26.39(21.67-32.78) |  | 24.34(18.55-30.60) | 0.00 |
| Buparlisib | 2.56(2.20-2.98) |  | 2.44(2.03-2.94) | 0.01 |
| AZD5363 | 19.73(15.64-25.37) |  | 16.54(12.49-24.51) | 0.00 |
| **Apoptosis regulation** | |  |  |  |
| MG.132 0.20(0.18-0.23) | |  | 0.19(0.16-0.21) | 0.00 |
| Pyridostatin 29.91(23.68-37.75) | |  | 26.93(19.06-36.31) | 0.00 |
| **Alkylating Agents** | |  |  |  |
| BPD.00008900 91.03(75.22-112.26) | |  | 86.98(65.00-109.17) | 0.02 |

**Abbreviations:** IC50: half maximal inhibitory concentration.
