# Supplementary material for: Identification and Validation of a Prognostic Model Based on Tumour Necrosis Factor‐Related mRNAs for Kidney Renal Clear Cell Carcinoma
Source: J Cell Mol Med. 2025 Jul 17;29(14):e70657. doi: 10.1111/jcmm.70657 (PMC12268967; doi:10.1111/jcmm.70657)
Supplement: Supplementary file 18 — Table S7. Antineoplastic drug sensitivity information (no obviously sensitive group). [file JCMM-29-e70657-s010.docx]

**Table S7** Antineoplastic drug sensitivity information (no obviously sensitive group).

| **Target pathway** | **Low-risk group** |  | **High-risk group** | **P-value** |
| --- | --- | --- | --- | --- |
|  | **IC50 (25%-75%)** |  | **IC50 (25%-75%)** |  |
| **WNT signaling** |  |  |  |  |
| AGI.6780 | 61.01(53.09-70.64) |  | 61.37(50.06-74.70) | 0.96 |
| **Unclassified** |  |  |  |  |
| Gallibiscoquinazole_1830 | 12.72(11.37-14.70) |  | 13.28(11.12-16.30) | 0.12 |
| ABT737 | 8.91(6.73-12.06) |  | 9.41(5.92-13.14) | 0.78 |
| Dihydrorotenone | 2.42(2.12-2.95) |  | 2.45(2.00-3.03) | 0.79 |
| **RTK signaling** |  |  |  |  |
| Staurosporine | 0.04(0.03-0.06) |  | 0.04(0.03-0.06) | 0.09 |
| Savolitinib | 13.94(11.40-16.21) |  | 13.57(10.30-17.48) | 0.6 |
| Crizotinib | 24.24(18.94-31.65) |  | 24.19(17.58-34.57) | 0.99 |
| AZD4547 | 17.36(14.17-21.40) |  | 17.05(13.18-24.17) | 0.94 |
| Foretinib | 2.54(2.08-3.44) |  | 2.42(1.94-3.44) | 0.12 |
| **Protein stability and degradation** | |  |  |  |
| BIBR.1532 139.57(110.56-166.23) | |  | 141.79(110.66-178.07) | 0.46 |
| ML323 86.22(73.70-99.51) | |  | 86.84(70.26-107.71) | 0.57 |
| LJI308 161.10(137.88-195.66) | |  | 157.30(123.22-194.37) | 0.07 |
| **PI3K/MTOR signaling** | |  |  |  |
| AZD6482 24.65(21.79-27.50) | |  | 23.94(20.62-28.62) | 0.59 |
| AZ6102 10.73(9.70-12.50) | |  | 10.90(9.64-12.96) | 0.37 |
| Uprosertib_1553 18.97(15.75-24.20) | |  | 18.88(14.90-25.92) | 0.83 |
| Alpelisib 35.98(24.14-52.64) | |  | 31.24(22.18-51.26) | 0.14 |
| Taselisib 7.87(5.47-11.64) | |  | 6.99(4.87-11.98) | 0.24 |
| CZC24832 159.94(129.74-192.53) | |  | 157.51(117.90-194.51) | 0.18 |
| AZD8186 25.81(21.72-30.56) | |  | 24.32(19.65-32.66) | 0.30 |
| LJI308 161.10(137.88-195.66) | |  | 157.30(123.22-194.37) | 0.07 |
| **Other, kinases** |  |  |  |  |
| WEHI.539 | 34.10(27.70-43.02) |  | 33.45(25.16-43.45) | 0.46 |
| UMI.77 | 14.60(11.09-18.04) |  | 15.17(11.13-20.69) | 0.07 |
| PAK_5339 | 10.53(9.30-12.30) |  | 10.66(8.95-13.06) | 0.9 |
| IGF1R_3801 | 5.00(3.62-7.20) |  | 4.55(3.30-7.09) | 0.22 |
| GSK343 | 16.53(14.26-19.30) |  | 16.35(13.21-19.44) | 0.29 |
| AT13148 | 34.14(26.81-49.17) |  | 35.42(25.41-53.63) | 0.73 |
| AMG.319 | 124.45(101.59-154.83) |  | 122.12(89.26-157.65) | 0.31 |
| Dasatinib | 4.85(3.56-7.61) |  | 4.59(2.94-7.00) | 0.18 |
| Ruxolitinib | 120.82(99.44-149.07) |  | 125.06(95.76-164.72) | 0.28 |
| Entospletinib | 40.75(33.74-49.18) |  | 37.73(30.38-48.66) | 0.10 |
| **Other** |  |  |  |  |
| CDK9_5576 | 0.66(0.52-0.85) |  | 0.64(0.48-0.83) | 0.21 |
| TAF1_5496 | 49.32(38.59-61.61) |  | 47.45(34.25-60.52) | 0.19 |
| P22077 | 83.98(65.55-106.11) |  | 84.17(65.77-118.36) | 0.16 |
| Dactinomycin_1811 | 0.08(0.07-0.11) |  | 0.08(0.06-0.011) | 0.11 |
| Zoledronate | 40.94(33.75-50.52) |  | 43.13(33.32-55.28) | 0.09 |
| **Mitosis** |  |  |  |  |
| Venetoclax | 8.67(7.27-10.49) |  | 8.25(6.64-10.83) | 0.18 |
| ZM447439 | 18.79(16.13-21.43) |  | 18.32(15.39-22.21) | 0.45 |
| Alisertib | 6.34(4.20-9.73) |  | 6.13(3.63-10.71) | 0.53 |
| Tozasertib | 17.76(14.91-21.82) |  | 17.79(14.54-23.64) | 0.75 |
| **IGF1R signaling** |  |  |  |  |
| GSK1904529A | 72.69(59.29-90.66) |  | 71.43(55.73-95.77) | 0.96 |
| Linsitinib | 43.85(34.93-55.67) |  | 41.94(31.86-54.53) | 0.09 |
| IGF1R_3801 | 5.00(3.62-7.20) |  | 4.55(3.30-7.09) | 0.22 |
| **Hormone-related** |  |  |  |  |
| Fulvestrant_1816 | 92.19(79.32-113.51) |  | 91.54(72.87-114.40) | 0.30 |
| **Genome integrity** |  |  |  |  |
| Niraparib | 73.59(54.87-101.00) |  | 69.14(47.39-103.22) | 0.15 |
| **ERK MAPK signaling** | |  |  |  |
| Ulixertinib_1908 15.00(12.48-19.54) | |  | 15.74(12.72-20.17) | 0.33 |
| Fludarabine 152.66(118.66-200.82) | |  | 147.74(105.11-207.54) | 0.36 |
| Ulixertinib_2047 8.90(7.23-11.65) | |  | 8.75(6.79-11.32) | 0.19 |
| Selumetinib 62.39(4793-80.78) | |  | 66.54(45.61-90.70) | 0.30 |
| OTX015 11.65(8.59-15.81) | |  | 10.58(7.53-16.91) | 0.22 |
| ERK_2440 14.15(11.32-18.05) | |  | 14.05(10.20-18.67) | 0.64 |
| Selumetinib 62.39(47.93-80.78) | |  | 66.54(45.61-90.70) | 0.30 |
| **DNA replication** |  |  |  |  |
| KRAS.G12C.Inhibitor.12 | 80.57(62.27-105.07) |  | 77.17(54.78-104.34) | 0.17 |
| Nelarabine | 397.97(319.48-495.22) |  | 423.19(316.45-554.00) | 0.09 |
| Picolinici.acid | 165.11(147.91-190.30) |  | 166.83(139.92-200.44) | 0.89 |
| ERK_2440 | 14.15(11.32-18.05) |  | 14.05(10.20-18.67) | 0.64 |
| Cyclophosphamide | 173.89(147.66-204.29) |  | 171.71(133.38-209.92) | 0.47 |
| Fludarabine | 152.66(118.66-200.82) |  | 147.74(105.11-207.54) | 0.36 |
| **Cytoskeleton** |  |  |  |  |
| I.BRD9 | 78.88(61.74-98.43) |  | 74.21(56.17-99.75) | 0.29 |
| PAK_5339 | 10.53(9.30-12.30) |  | 10.66(8.95-13.06) | 0.90 |
| **Chromatin other** |  |  |  |  |
| MN.64 | 111.48(94.04-132.75) |  | 108.88(91.98-134.98) | 0.56 |
| AZD4547 | 17.36(14.17-21.40) |  | 17.05(13.18-24.17) | 0.94 |
| PFI3 | 183.35(163.78-216.07) |  | 190.02(155.70-226.56) | 0.82 |
| EPZ5676 | 245.02(206.34-306.56) |  | 255.49(196.02-327.87) | 0.39 |
| GSK343 | 16.53(14.26-19.30) |  | 16.35(13.21-19.44) | 0.29 |
| GSK591 | 94.52(80.02-119.85) |  | 92.01(72.68-120.04) | 0.13 |
| Dinaciclib | 0.06(0.05-0.08) |  | 0.06(0.04-0.08) | 0.18 |
| CDK9_5576 | 0.66(0.52-0.85) |  | 0.64(0.48-0.83) | 0.21 |
| Entospletinib | 40.75(33.74-49.18) |  | 37.73(30.38-48.66) | 0.10 |
| **EGFR signaling** |  |  |  |  |
| ML323 | 86.22(73.70-99.51) |  | 86.84(70.26-107.71) | 0.57 |
| Foretinib | 2.54(2.08-3.44) |  | 2.42(1.94-3.57) | 0.12 |
| JAK1_8709 | 62.46(52.96-76.52) |  | 61.37(50.30-79.44) | 0.65 |
| Acetalax | 130.34(99.26-175.75) |  | 135.57(96.41-193.73) | 0.49 |
| **Metabolism** |  |  |  |  |
| GSK591 | 94.52(80.02-119.85) |  | 92.01(72.68-120.04) | 0.13 |
| Dihydrorotenone | 2.42(2.12-2.95) |  | 2.45(2.00-3.03) | 0.79 |
| **Cell cycle** |  |  |  |  |
| AZD8186 | 25.81(21.72-30.56) |  | 24.32(19.65-32.66) | 0.30 |
| **Apoptosis regulation** |  |  |  |  |
| JQ1 | 15.04(13.98-16.20) |  | 15.02(13.84-16.47) | 0.59 |
| AZD5991 | 71.81(47.67-113.36) |  | 73.30(41.33-128.17) | 0.92 |
| Navitoclax | 6.96(4.49-10.52) |  | 6.47(4.05-11.08) | 0.25 |
| AZD5991 | 71.81(47.67-113.36) |  | 73.30(41.33-128.17) | 0.92 |
| Venetoclax | 8.67(7.27-10.49) |  | 8.25(6.64-10.83) | 0.18 |
| ABT737 | 8.91(6.73-12.06) |  | 9.41(5.92-13.14) | 0.78 |
| **ABL signaling** |  |  |  |  |
| Nilotinib | 37.57(27.09-48.83) |  | 35.94(23.73-50.29) | 0.39 |

**Abbreviations:** IC50: half maximal inhibitory concentration.
